# Supplementary material for: Microenvironmental modulation breaks intrinsic pH limitations of nanozymes to boost their activities
Source: Nat Commun. 2024 Dec 30;15:10861. doi: 10.1038/s41467-024-55163-4 (PMC11686145; doi:10.1038/s41467-024-55163-4)
Supplement: Supplementary file 1 — Supplementary Information [file 41467_2024_55163_MOESM1_ESM.pdf]

Supporting Information

Microenvironmental modulation breaks intrinsic pH limitations of nanozymes to boost their activities

Tong Li[1, #], Xiaoyu Wang[2, #], Yuting Wang[1], Yihong Zhang[1], Sirong Li[1], Wanling Liu[1], Shujie Liu[1], Yufeng Liu[1], Hang Xing[3], Ken-ichi Otake[4], Susumu Kitagawa[4], Jiangjiexing Wu[1, 5, *], Hao Dong[2, 6, 7, 8, 9, *], Hui Wei[1, 6, 9, *]

[1] College of Engineering and Applied Sciences, Nanjing National Laboratory of Microstructures, Jiangsu Key Laboratory of Artificial Functional Materials, Nanjing University, Nanjing, Jiangsu, China

[2] Kuang Yaming Honors School, Nanjing University, Nanjing, Jiangsu, China

[3] Institute of Chemical Biology and Nanomedicine, State Key Laboratory of Chemo/Biosensing and Chemometrics, College of Chemistry and Chemical Engineering, Hunan University, Changsha, Hunan, China

[4] Institute for Integrated Cell-Material Sciences (WPI-iCeMS), Kyoto University, Sakyo-ku, Japan

[5] School of Marine Science and Technology, Tianjin University, Tianjin, 300072, China

[6] State Key Laboratory of Analytical Chemistry for Life Science, Nanjing University, Nanjing, Jiangsu, China

[7] Institute for Brain Sciences, Nanjing University, Nanjing, Jiangsu, China

[8] Engineering Research Centre of Protein and Peptide Medicine of Ministry of Education, Nanjing University, Nanjing, Jiangsu, China

[9] Chemistry and Biomedicine Innovation Centre (ChemBIC), ChemBioMed Interdisciplinary Research Centre at Nanjing University, Nanjing University, Nanjing, Jiangsu, China

*e-mail: weihui@nju.edu.cn (Hui Wei); donghao@nju.edu.cn (Hao Dong); wujiangjiexing2007@126.com (Jiangjiexing Wu)

Table of Contents

Supplementary Methods

1. Reagents
2. Calibration of the commercial H_2O_2 (30 wt%)
3. Fabrication of Fe-TCPP
4. Fabrication of PCN-222 NPs and PCN-222@PAA NPs
5. pH-dependent stability of PCN-222-Fe@PAA NPs
6. pH-dependent oxidation of TMB by Ce^{4+} ions
7. Fabrication of other nanozymes
8. Fabrication of MOF-808 NPs and MOF-808@PEI/PEGDE NPs
9. Fabrication of other Zr-MOF NPs and Zr-MOF@PEI/PEGDE NPs
10. Titration of PAA
11. Hydrolase-like activity measurements
12. Activity measurement of several oxidases
13. Oxidase/MOF nanozyme cascade reactions
14. Biomolecules sensing
15. Chirality recognition of natural amino acid
16. Chirality recognition of non-natural amino acid
17. Characterizations

Supplementary Figures

Supplementary Fig. 1. Charge transfer system containing TMB and Ce^{4+} ions at different pHs.
Supplementary Fig. 2. Bjerrum plot of TMB and its protonated species.
Supplementary Fig. 3. Length and width distribution of PCN-222-Fe NPs and PCN-222-Fe@PAA NPs.
Supplementary Fig. 4. XRD patterns of PCN-222-Fe NPs and simulated PCN-222.
Supplementary Fig. 5. Influence of PAA incubation on linkers leaching in PCN-222-Fe NPs and PCN-222 NPs.
Supplementary Fig. 6. Zeta potentials of PCN-222-Fe NPs and PCN-222-Fe@PAA NPs.
Supplementary Fig. 7. Measurement of the molar concentration of HRP solution.
Supplementary Fig. 8. H_2O_2 calibration.
Supplementary Fig. 9. (a) N_2 isotherms and (b) pore size distributions of PCN-222-Fe NPs and PCN-222-Fe@PAA NPs.
Supplementary Fig. 10. Zr 3d XPS spectra of PCN-222-Fe NPs and PCN-222-Fe@PAA NPs.
Supplementary Fig. 11. TG curves of PAA, PCN-222-Fe NPs, and PCN-222-Fe@PAA NPs.
Supplementary Fig. 12. pH-dependent operational stability of PCN-222-Fe NPs.
Supplementary Fig. 13. Improved activity for a fluorescent substrate, Amplex Red, at physiological pH.
Supplementary Fig. 14. Organic linkers in PCN-222-Fe NPs serving as catalytically active sites.
Supplementary Fig. 15. Iron porphyrin linkers serving as catalytically active sites.
Supplementary Fig. 16. Activity decrease of PCN-222-Fe NPs induced by PAA confinement under acidic condition.

Supplementary Fig. 17. PAA incubation achieves the improved peroxidase-like activity of PCN-222-Fe NPs.
Supplementary Fig. 18. Effect of PAA on the bulk pH of Tris buffer.
Supplementary Fig. 19. Lineweaver-Burk plots of reciprocal velocities against reciprocal H_2O_2 concentrations.
Supplementary Fig. 20. Influence of NaCl concentration on the peroxidase-like activity of PCN-222-Fe@PAA NPs.
Supplementary Fig. 21. XRD patterns of PCN-222 NPs before and after PAA modification, and simulated PCN-222.
Supplementary Fig. 22. Zeta potentials of PCN-222 NPs and PCN-222@PAA NPs.
Supplementary Fig. 23. Influence of protons addition on the light absorption of PCN-222 NPs.
Supplementary Fig. 24. Absorption spectra of PCN-222 NPs and PCN-222@PAA NPs.
Supplementary Fig. 25. Absorption spectra of TCPP DMF solution with addition of protons.
Supplementary Fig. 26. Normalised fluorescence intensity of PCN-222 NPs at 660 nm at different pHs.
Supplementary Fig. 27. Fluorescence emission spectra of PCN-222 NPs and PCN-222@PAA NPs.
Supplementary Fig. 28. Influence of PAA concentration on the microenvironmental pH decrease.
Supplementary Fig. 29. Influence of PAA molecular weight on the microenvironmental pH decrease.
Supplementary Fig. 30. Microenvironmental pH decrease enabled by PAA confinement after thorough washing.
Supplementary Fig. 31. Effect of other polymers on the activity of PCN-222-Fe NPs.
Supplementary Fig. 32. Deprotonated PAA does not increase the peroxidase-like activity of PCN-222-Fe NPs.
Supplementary Fig. 33. Titration curve of PAA.
Supplementary Fig. 34. Equilibrium of the number of Tris and water in a PAA-modified channel in MD simulations.
Supplementary Fig. 35. Representative TEM images and hydrodynamic size distributions of (a) Fe_3O_4 NPs, (b) graphene oxide, (c) NH_2-MIL-88B NPs, and (d) Pt NPs obtained from one batch of NPs.
Supplementary Fig. 36. Activity comparison with other nanozymes.
Supplementary Fig. 37. PAA modification increases the peroxidase-like activity of PCN-222-Fe NPs at pH 7.4.
Supplementary Fig. 38. Activity measurement of urate oxidase (UOx) and one-pot oxidase/MOF nanozyme cascade reaction.
Supplementary Fig. 39. Activity measurement of four representative oxidases and one-pot oxidase/MOF nanozyme cascade reactions.
Supplementary Fig. 40. Biomolecules sensing enabled by oxidase/PCN-222-Fe@PAA cascade reactions.
Supplementary Fig. 41. Activity measurement of D-amino acid oxidase (DAAO).
Supplementary Fig. 42. Gelation of PEI and PEGDE in ethanol.
Supplementary Fig. 43. (a) N_2 isotherms and (b) pore size distributions of MOF-808 NPs and MOF-808@PEI/PEGDE NPs.
Supplementary Fig. 44. TG curves of MOF-808 NPs and MOF-808@PEI/PEGDE NPs.

Supplementary Fig. 45. Representative SEM images and XRD patterns of MOF-808@(PEI/PEGDE)$_n$ NPs.
Supplementary Fig. 46. Influence of pH on the absorption of pNP.
Supplementary Fig. 47. Catalytic hydrolysis of pNPP by MOF-808@(PEI/PEGDE)$_n$ NPs.
Supplementary Fig. 48. pH indication by phenol red.
Supplementary Fig. 49. pH indication of the supernatant of MOF-808@(PEI/PEGDE)$_n$ NPs.
Supplementary Fig. 50. UV–vis spectra of pNPP solutions before and after 20-min incubation with free PEI.
Supplementary Fig. 51. Improved hydrolase-like activity of other Zr-MOF NPs through PEI confinement.
Supplementary Fig. 52. pH indication of the supernatant of Zr-MOF NPs by phenol red.
Supplementary Fig. 53. Influence of PEI incubation on TCPP leaching in PCN-222 NPs.
Supplementary Fig. 54. PEI confinement increases the hydrolase-like activity of PCN-222 NPs.
Supplementary Fig. 55. PEI confinement achieves alkaline microenvironment.

Supplementary Tables

Supplementary Table 1. Summary of BET surface areas of the nanozymes.
Supplementary Table 2. The calculated binding strength between PAA and different ligands.

Supplementary References

Supplementary Methods

1. Reagents

Ferrous chloride tetrahydrate ($FeCl_2•4H_2O$, ⩾98%), ceric ammonium nitrate ($(NH_4)_2Ce(NO_3)_6$, ≥99%) propionic acid (≥99.5%), N,N-dimethylformamide (DMF, ≥99.5%), pyrrole (99%), hydrochloric acid (HCl, 36%~38%), trichloromethane ($CHCl_3$, ≥99%), methanol (CH_3OH, ≥99.5%), ethanol (≥99.7%), tetrahydrofuran (THF, ≥99.5%), potassium hydroxide (KOH, ≥85%), benzoic acid (BA, ≥99.5%), hydrogen peroxide (H_2O_2, 30 wt% in water), tris(hydroxymethyl)methyl aminomethane (Tris, ≥99%), potassium chloride (KCl, ≥99.5%), sodium chloride (NaCl, ≥99.5%), sodium sulfate (Na_2SO_4, ≥99%), glucose (AR), ferric chloride hexahydrate ($FeCl_3•6H_2O$, ≥99%), formic acid (90%), acetic acid (≥99.5%), dichloroacetic acid (DCA, ≥99%), sodium acetate (≥99%), ethylene glycol (≥99.5%), chloroplatinic acid hexahydrate ($H_2PtCl_6•6H_2O$, AR), and acetone (≥99.5%) were purchased from Sinopharm Chemical Reagent Co., Ltd. Methyl p-formylbenzoate (≥98%), 3,3',5,5'-tetramethylbenzidine dihydrochloride (TMB, ≥98%), poly(acrylic acid) with M_w = 2 kDa (PAA), 1,3,5-benzenetricarboxylic acid (also named trimesic acid, H_3BTC, ≥98%), poly(ethylene imine) (PEI, M_w = 10 kDa, ≥99%), polyvinyl pyrrolidone (PVP, M_w = 24 kDa), HOOC-PEG-COOH (M_w = 2 kDa), poly(ethylene glycol) diglycidyl ether (PEGDE, M_n = 500 Da), 4,4'-biphenyldicarboxylic acid (≥98%) were obtained from Aladdin. Zirconium oxychloride octahydrate ($ZrOCl_2•8H_2O$, 99%) was purchased from Energy Chemical. Zirconium tetrachloride ($ZrCl_4$, ≥98%) was bought from J&K Scientific. Poly(sodium 4-styrenesulfonate) (PSS, M_w = 70 kDa) was obtained from Acros. Poly(allylamine hydrochloride) (PAH, M_w = 120 kDa~140 kDa) and phenol red were purchased from Alfa Aesar. Amplex Red was purchased from Beyotime. Tetrakis(4-carboxyphenyl)porphyrin (TCPP, >97%) was obtained from TCI. Urate oxidase (UOx) from arthrobacter protophormiae (≥10 U/mg), choline chloride (98%), D-alanine (98%), D-valine (98%), D-proline (99%), and D-isoleucine (98%) was bought from Meryer. Lactate Oxidase (LOx, ≥20 U/mg), and choline oxidase (COx, 8–20 U/mg) was bought from Macklin. Alcohol oxidase (AOx, ≥7 U/mg), D-amino acid oxidase (DAAO, 8 U/mg), and poly(acrylic acid) (M_w = 450 kDa, termed as $PAA_{450\ kDa}$) was bought from Shanghai yuanye Bio-Technology. L-alanine (98%), L-valine (96%), L-proline (97%), and L-isoleucine (98%) were bought from Tianjin Heowns. 3-Chloro-L-alanine (98%), 3-chloro-D-alanine (95%), L-2-Aminobutyric acid (97%), D-2-Aminobutyric acid (97%), L-Propargylglycine (97%), D-Propargylglycine (95%), L-Cyclohexylglycine (97%), and D-Cyclohexylglycine (97%), 4,4',4'',4'''-(pyrene-1,3,6,8-tetrayl)tetrabenzoic acid (TBAPy, 97%), and 4-nitrophenyl phosphate disodium salt (pNPP, 98%) were bought from Bidepharm.

2. Calibration of the commercial H_2O_2 (30 wt%)

The concentration of the commercial H_2O_2 was calibrated according to a reported procedure.[2] Before H_2O_2 calibration, the molar concentration of HRP was identified by its absorbance at 403 nm with a coefficient of $1 \times 10^5\ M^{-1}•cm^{-1}$.[2] The commercial H_2O_2 concentration was stated to be 9.9 M. It was diluted to several stated concentrations. Forty microliters of 10 mM ABTS, 40 μL of 2 μM HRP, and 40 μL of the diluted H_2O_2 solution were added sequentially into 1.88 mL of 0.2 M Tris buffer (pH = 7.4). The absorbance changes at 600 nm were recorded. By using a coefficient of $ABTS^{+•}$ at 600 nm of $1.1 \times 10^4\ M^{-1}•cm^{-1}$, the H_2O_2 concentrations were calculated, which should be half of the $ABTS^{+•}$ concentrations. The calculated concentrations were plotted against the stated concentrations and fitted linearly with a slope of 0.838. Hence, the concentration of the commercial

H_2O_2 is 8.3 M.

3. Fabrication of Fe-TCPP

Typically,[1] 14 g methyl p-formylbenzoate was dissolved in 200 mL of propionic acid in a 500 mL flask. Pyrrole with a volume of 6 mL was added dropwise into the flask, which was further refluxed for 12 h. After cooling down to room temperature, purple precipitates (5,10,15,20-tetrakis(4-methoxycarbonylphenyl)porphyrin, TPP-COOMe) were collected by suction filtration and washed with methanol, ethyl acetate and tetrahydrofuran. After drying in vacuum, 854 mg TPP-COOMe was added into 100 mL of DMF in a 250 mL flask, followed by the addition of 2.5 g $FeCl_2•4H_2O$. The flask was further refluxed for 6 h. After cooling down to room temperature, brown precipitates were collected through suction-filtration after addition of 150 mL of water. After washing with water and drying under vacuum, the brown solids were dissolved in $CHCl_3$ and washed with 1.0 M HCl and water for three times. The organic layer was dried by Na_2SO_4, and $CHCl_3$ was further removed by rotary evaporation leaving brown solids in the bottom of the flask. Then, 25 mL of methanol and 25 mL of THF were added into the flask to dissolve the solids, followed by the addition of 25 mL of water containing 2.63 g KOH. The flask was refluxed for 12 h. After cooling down to room temperature, methanol and TFH were removed by rotary evaporation. Water with a volume of 25 mL was supplemented to the flask to fully dissolve the solids under heating. Then, 50 mL of 1.0 M HCl was added to obtain the brown precipitates, which were further collected by suction-filtration and washed with water for several times. After drying in vacuum, Fe-TCPP was obtained as brown solids.

4. Fabrication of PCN-222 NPs and PCN-222@PAA NPs

Typically,[3] 150 mg $ZrOCl_2•8H_2O$ and 26 mg TCPP were dissolved in 64 mL of DMF, followed by the addition of 1.6 mL of DCA. The solution was sonicated for 5 min and transferred into a Teflon-lined autoclave. It was then heated at 130 °C for 18 h in an oven. After cooling to room temperature, PCN-222 NPs were obtained and washed with DMF several times. The PCN-222 NPs were further dispersed in 10 mL of DMF, and 50 μL of concentrated HCl was introduced, followed by heating at 80 °C for 12 h. The PCN-222 NPs were finally washed with DMF several times, dispersed in DMF, and stored at 4 °C. PCN-222@PAA NPs were obtained by incubating 0.5 mg/mL PCN-222 NPs within 1.0 mg/mL PAA for 30 min, followed by washing with water three times.

To measure the fluorescent spectra of NPs, 200 μL of 20 μg/mL PCN-222 NPs or PCN-222@PAA NPs were added to a 96-well plate. The fluorescent spectra were measured by using a microplate reader with an excitation wavelength at 420 nm.

5. pH-dependent stability of PCN-222-Fe@PAA NPs

Typically, 10 μL of 0.25 mg/mL PCN-222-Fe@PAA NPs was mixed with 10 μL of water or 10 μL of 10 mM buffer solutions at pHs 3–9. After 1-h incubation at room temperature, 460 μL of 200 mM Tris buffer with a pH of 7.4 was added, followed by the introduction of 10 μL of 10 mM TMB and 10 μL of 10 mM H_2O_2 to initiate reactions. After 2-min reaction, 200 μL of the supernatant was collected through 5-min centrifugation. Optical density at 652 nm ($OD_{652\ nm}$) was recorded using a microplate reader.

6. pH-dependent oxidation of TMB by Ce^{4+} ions

Typically, 10 μL of 5 mM $(NH_4)_2Ce(NO_3)_6$ was added into 980 μL of 200 mM buffer solutions at pHs 3–8, followed by the addition of 5 mM TMB. After 10-min reaction, 200 μL of the reaction solutions was taken, and $OD_{652\ nm}$ and optical density at 450 nm ($OD_{450\ nm}$) were recorded using a microplate reader.

7. Fabrication of other nanozymes

For Fe_3O_4 NPs, in a typical procedure,[4] 410 mg $FeCl_3$•$6H_2O$ and 1.9 g sodium acetate were added to 20 mL of ethylene glycol. The above mixture was stirred for 30 min at room temperature, added to a Teflon-lined autoclave, and heated at 200 °C for 12 h. After cooling down to room temperature, the suspension was transferred into a tube. Fe_3O_4 NPs were collected at the bottom of the tube by a magnet, and the supernatant was removed. Ethanol with a volume of about 20 mL was added and Fe_3O_4 NPs were redispersed by shaking. A magnet was further used to collect Fe_3O_4 NPs, and the supernatant was removed. Such a washing step was repeated for three times. Finally, Fe_3O_4 NPs were dried under vacuum.

For NH_2-MIL-88B NPs, In a typical procedure,[5] 18 mg 2-aminoterephthalic acid and 27 mg $FeCl_3$•$6H_2O$ were dissolved in 10 mL of ethanol. The solution was transferred to a 20-mL vial and heated at 40 °C for 1.5 h in a water bath. After the reaction, the NH_2-MIL-88B NPs were collected by centrifugation at 10,280 × g for 10 min. After washing three times with ethanol and three times with acetone, the NPs were dried under vacuum.

For Pt NPs, In a typical procedure,[6] 266 mg PVP (M_w = 24 kDa) was dissolved into 90 mL of methanol in a 250-mL flask. Then, 10 mL of 50 mM H_2PtCl_6 was introduced. The above solution was refluxed for 3 h. After the reaction and cooling, the methanol was evaporated. The Pt NPs were purified with acetone, redispersed in water, and stored at 4 °C. The mass concentration of Pt NPs was identified by an inductively coupled plasma mass spectrometer.

8. Fabrication of MOF-808 NPs and MOF-808@PEI/PEGDE NPs

Typically,[7] 70 mg H_3BTC was dissolved in 10 mL of DMF first, and then 323 mg $ZrOCl_2$•$8H_2O$ was added and dissolved, followed by the introduction of 10 mL of formic acid. The solution was sonicated several minutes and heated at 100 °C for one day in an oven. The formed powder was further washed several times using DMF and dispersed in 20 mL of DMF with the addition of 1 mL of 8 M HCl. The suspension was heated at 90 °C for 12 h. Finally, the MOF-808 NPs were obtained after washing with DMF several times and stored in DMF at 4 °C.

For the preparation of MOF-808@PEI/PEGDE NPs, typically, 10 mg MOF-808 in DMF was centrifuged and washed several times using ethanol. Then, it was dispersed in 1 mL of 20 mg/mL PEI ethanol solution. The suspension was incubated at 37 °C for 1 h in a water bath. After that, the suspension was centrifuged and the supernatant was removed, followed by the addition of 1 mL of 20 mg/mL PEGDE ethanol solution. The suspension was further incubated at 37 °C for another 1 h. The MOF-808@PEI/PEGDE NPs were obtained after washing with ethanol at least four times and finally dispersed in 1 mL of ethanol. The MOF-808@PEI/PEGDE NPs were stored at 4 °C. When the process of PEI/PEGDE incubation was employed twice, three times, four times, and five times, MOF-808@$(PEI/PEGDE)_n$ NPs with n = 2, 3, 4, and 5 were obtained.

9. Fabrication of other Zr-MOF NPs and Zr-MOF@PEI/PEGDE NPs

For UiO-66 NPs,[8] 125 mg $ZrCl_4$ was dissolved into 5 mL of DMF, followed by the addition of 1 mL of concentrated HCl. Then, 123 mg terephthalic acid and 10 mL of DMF were further introduced into the above solution, which was sonicated for several minutes and heated at 80 °C for 12 h. The UiO-66 NPs were washed several times using DMF and stored in DMF at 4 °C.

For NH_2-UiO-66 NPs,[8] the synthesis process was the same as that for UiO-66 NPs except that 134 mg 2-aminoterephthalic acid was employed.

For UiO-67 NPs,[8] the synthesis process was the same as that for UiO-66 NPs except that 67 mg $ZrCl_4$, 90 mg 4,4'-biphenyldicarboxylic acid, and 0.5 mL of concentrated HCl were employed.

For NU-1000 NPs,[9] 40 mg $ZrOCl_2$•$8H_2O$ and 900 mg BA were dissolved in 4 mL of DMF in a 25-mL vial. TBAPy (10 mg) was dispersed in 4 mL of DMF in a 25-mL vial. The two vials were heated at 120 °C for 1 h. After cooling to room temperature, the solutions were mixed together with sonication for several minutes. After reaction at 120 °C for 1 h, NU-1000 NPs were washed with DMF several times and dispersed in 5 mL of DMF with the addition of 150 μL of 8 M HCl. The suspension was heated at 80 °C for 12 h. After cooling to room temperature, NU-1000 NPs were washed with DMF several times and stored in DMF at 4 °C.

For Zr-MOF@PEI/PEGDE NPs, the preparation process was the same as that for MOF-808@PEI/PEGDE NPs.

10. Titration of PAA

To determine the apparent pK_a of PAA, 288 mg PAA (4 mmol carboxyl groups) was first dissolved into 40 mL of water in a 100-mL beaker under stirring. The pH of the PAA solution was monitored every time when 40 μL of 1 M NaOH (0.01 N) was added. The titration ended with the accumulative addition of 1 N NaOH. The apparent pK_a was the pH when 0.5 N NaOH was introduced.

11. Hydrolase-like activity measurements

For substrate pNPP, 36 μL of 10 mg/mL MOF-808 NPs (or MOF-808@PEI/PEGDE NPs) was added to 1.728 mL of water, followed by the introduction of 36 μL of 100 mM pNPP to start the reaction. After a 20-min reaction at room temperature, the MOF NPs were removed by centrifugation. Then, 1 mL of the supernatant was added to 1 mL of 100 mM phosphate buffer at pH 7.0, which was used for absorption measurement by using a spectrophotometer.

12. Activity measurement of several oxidases

For urate oxidase (UOx), typically, 20 μL of UOx (5 mg/mL) was added into 1.96 mL of 0.2 M buffer with a pH of 4.0 or pH of 7.4. Then, 20 μL of 5 mM uric acid was introduced to initiate the reaction. The absorbance change at 292 nm ($A_{292\ nm}$) was immediately monitored which reflected the consumption of uric acid. UOx activity at pH 4.0 or pH 7.4 was demonstrated by the initial consumption rate of uric acid.

For lactate oxidase (LOx), typically, 2.5 μL of LOx (50 U/mL) was added into 200 μL of 0.2 M buffer with a pH of 4.0 or pH of 7.4. Then, 4 μL of 50 mM lactic acid was introduced to initiate the reaction. After 10-min reaction, 20 μL of the reaction solution was added into a 96-well plate, followed by the introduction of 200 μL of 0.2 M Tris buffer (pH 7.4) containing 5 μg/mL PCN-222-Fe@PAA NPs and 50 μM Amplex Red. The fluorescence intensity change at 585 nm ($I_{585\ nm}$) was immediately monitored which reflected the amount of produced H_2O_2 catalysed by LOx. The

excitation wavelength was 560 nm. LOx activity at pH 4.0 or pH 7.4 was demonstrated by monitoring the velocity of $I_{585\ nm}$.

For choline oxidase (COx), typically, 2.5 μL of COx (50 U/mL) was added into 200 μL of 0.2 M buffer with a pH of 4.0 or pH of 7.4. Then, 2.5 μL of 50 mM choline chloride was introduced to initiate the reaction. After 10-min reaction, 20 μL of the reaction solution was added into a 96-well plate, followed by the introduction of 200 μL of 0.2 M Tris buffer (pH 7.4) containing 10 μg/mL PCN-222-Fe@PAA NPs and 50 μM Amplex Red. The fluorescence intensity change at 585 nm ($I_{585\ nm}$) was immediately monitored. The excitation wavelength was 560 nm. COx activity at pH 4.0 or pH 7.4 was demonstrated by monitoring the velocity of $I_{585\ nm}$.

For alcohol oxidase (AOx), typically, 2.5 μL of AOx (50 U/mL) was added into 200 μL of 0.2 M buffer with a pH of 4.0 or pH of 7.4. Then, 2.5 μL of 200 mM ethanol was introduced to initiate the reaction. After 20-min reaction, 10 μL of the reaction solution was added into a 96-well plate, followed by the introduction of 200 μL of 0.2 M Tris buffer (pH 7.4) containing 10 μg/mL PCN-222-Fe@PAA NPs and 50 μM Amplex Red. The fluorescence intensity change at 585 nm ($I_{585\ nm}$) was immediately monitored. The excitation wavelength was 560 nm. AOx activity at pH 4.0 or pH 7.4 was demonstrated by monitoring the velocity of $I_{585\ nm}$.

For D-amino acid oxidase (DAAO), typically, 2.5 μL of DAAO (50 U/mL) was added into 100 μL of 0.2 M buffer with a pH of 4.0 or pH of 7.4. Then, 2.5 μL of 200 mM D-alanine was introduced to initiate the reaction. After 10-min reaction, 10 μL of the reaction solution was added into a 96-well plate, followed by the introduction of 200 μL of 0.2 M Tris buffer (pH 7.4) containing 10 μg/mL PCN-222-Fe@PAA NPs and 50 μM Amplex Red. The fluorescence intensity change at 585 nm ($I_{585\ nm}$) was immediately monitored. The excitation wavelength was 560 nm. DAAO activity at pH 4.0 or pH 7.4 was demonstrated by monitoring the velocity of $I_{585\ nm}$.

13. Oxidase/MOF nanozyme cascade reactions

For UOx/MOF nanozyme cascade reaction, typically, 40 μL of 0.25 mg/mL PCN-222-Fe NPs or PCN-222-Fe@PAA NPs, 40 μL of 10 mM TMB, and 40 μL of 5 mM uric acid were added into 1.84 mL of buffer with a pH of 4.0 or pH of 7.4. Then, 40 μL of 5 mg/mL UOx was introduced to initiate the cascade reaction. The absorbance change at 652 nm ($A_{652\ nm}$) was immediately monitored.

For LOx/MOF nanozyme cascade reaction, typically, 40 μL of 0.25 mg/mL PCN-222-Fe NPs or PCN-222-Fe@PAA NPs, 40 μL of 10 mM TMB, and 40 μL of 50 mM lactic acid were added into 1.87 mL of buffer with a pH of 4.0 or pH of 7.4. Then, 10 μL of LOx (50 U/mL) was introduced to initiate the cascade reaction. The absorbance change at 652 nm ($A_{652\ nm}$) was immediately monitored.

For COx/MOF nanozyme cascade reaction, typically, 40 μL of 0.25 mg/mL PCN-222-Fe NPs or PCN-222-Fe@PAA NPs, 40 μL of 10 mM TMB, and 40 μL of 50 mM choline chloride were added into 1.86 mL of buffer with a pH of 4.0 or pH of 7.4. Then, 20 μL of COx (50 U/mL) was introduced to initiate the cascade reaction. The absorbance change at 652 nm ($A_{652\ nm}$) was immediately monitored.

For AOx/MOF nanozyme cascade reaction, typically, 40 μL of 0.25 mg/mL PCN-222-Fe NPs or PCN-222-Fe@PAA NPs, 40 μL of 10 mM TMB, and 40 μL of 200 mM ethanol were added into 1.87 mL of buffer with a pH of 4.0 or pH of 7.4. Then, 10 μL of AOx (50 U/mL) was introduced to initiate the cascade reaction. The absorbance change at 652 nm ($A_{652\ nm}$) was immediately monitored.

For DAAO/MOF nanozyme cascade reaction, typically, 40 μL of 0.25 mg/mL PCN-222-Fe NPs or PCN-222-Fe@PAA NPs, 40 μL of 10 mM TMB, and 40 μL of 200 mM D-alanine were

added into 1.87 mL of buffer with a pH of 4.0 or pH of 7.4. Then, 10 μL of DAAO (50 U/mL) was introduced to initiate the cascade reaction. The absorbance change at 652 nm ($A_{652\ nm}$) was immediately monitored.

14. Biomolecules sensing

For uric acid sensing, typically, 8 μL of 5 mg/mL UOx, 8 μL of 0.25 mg/mL PCN-222-Fe@PAA NPs, and 8 μL of 10 mM TMB were added into 368 μL of 0.2 M Tris buffer with a pH of 7.4. Then, 8 μL of uric acid with varying concentrations was introduced. After 20-min reaction, 200 μL of the reaction solution was taken into a 96-well plate, and the optical density at 652 nm ($OD_{652\ nm}$) was monitored. The concentration of uric acid ranged from 10 μM to 50 μM. For selectivity study, 250 μM interferences were employed.

For lactic acid sensing, typically, 4 μL of LOx (50 U/mL), 8 μL of 0.25 mg/mL PCN-222-Fe@PAA NPs, and 8 μL of 10 mM TMB were added into 372 μL of 0.2 M Tris buffer with a pH of 7.4. Then, 8 μL of lactic acid with varying concentrations was introduced. After 20-min reaction, 200 μL of the reaction solution was taken into a 96-well plate, and the optical density at 652 nm ($OD_{652\ nm}$) was monitored. The concentration of lactic acid ranged from 25 μM to 100 μM. For selectivity study, 500 μM interferences were employed.

For choline sensing, typically, 12 μL of COx (50 U/mL), 12 μL of 0.25 mg/mL PCN-222-Fe@PAA NPs, and 8 μL of 10 mM TMB were added into 360 μL of 0.2 M Tris buffer with a pH of 7.4. Then, 8 μL of choline chloride with varying concentrations was introduced. After 40-min reaction, 200 μL of the reaction solution was taken into a 96-well plate, and the optical density at 652 nm ($OD_{652\ nm}$) was monitored. The concentration of choline ranged from 10 μM to 50 μM. For selectivity study, 250 μM interferences were employed.

For ethanol sensing, typically, 12 μL of AOx (50 U/mL), 12 μL of 0.25 mg/mL PCN-222-Fe@PAA NPs, and 8 μL of 10 mM TMB were added into 360 μL of 0.2 M Tris buffer with a pH of 7.4. Then, 8 μL of ethanol with varying concentrations was introduced. After 40-min reaction, 200 μL of the reaction solution was taken into a 96-well plate, and the optical density at 652 nm ($OD_{652\ nm}$) was monitored. The concentration of ethanol ranged from 50 μM to 200 μM. For selectivity study, 1000 μM interferences were employed.

15. Chirality recognition of natural amino acid

Typically, 4 μL of DAAO (50 U/mL), 8 μL of 0.25 mg/mL PCN-222-Fe@PAA NPs, and 8 μL of 10 mM TMB were added into 372 μL of 0.2 M Tris buffer with a pH of 7.4. Then, 8 μL of 200 mM D-amino acid including D-alanine, D-valine, D-proline, and D-isoleucine or L-amino acid including L-alanine, L-valine, L-proline, and L-isoleucine was introduced to initiate the reaction. After 30-min reaction, 200 μL of the reaction solution was taken into a 96-well plate, and the optical density at 652 nm ($OD_{652\ nm}$) was monitored. For anti-interference study, 4 mM interferences were employed.

For fluorescent method, typically, 2.5 μL of DAAO (50 U/mL), 8 μL of 0.25 mg/mL PCN-222-Fe@PAA NPs, and 10 μL of 2 mM Amplex Red were added into 372 μL of 0.2 M Tris buffer with a pH of 7.4. Then, 8 μL of 200 mM D-amino acid including D-alanine, D-valine, D-proline, and D-isoleucine or L-amino acid including L-alanine, L-valine, L-proline, and L-isoleucine was introduced to initiate the reaction. After 10-min reaction, 200 μL of the reaction solution was taken into a 96-well plate, and the fluorescence intensity change at 585 nm ($I_{585\ nm}$) was immediately monitored. The excitation wavelength was 560 nm.

16. Chirality recognition of non-natural amino acid

Typically, 2.5 μL of DAAO (50 U/mL), 8 μL of 0.25 mg/mL PCN-222-Fe@PAA NPs, and 10 μL of 2 mM Amplex Red were added into 364 μL of 0.2 M Tris buffer with a pH of 7.4. Then, 16 μL of 100 mM D-amino acid or L-amino acid was introduced to initiate the reaction. After 10-min reaction, 200 μL of the reaction solution was taken into a 96-well plate, and the fluorescence intensity change at 585 nm ($I_{585\ nm}$) was immediately monitored. The excitation wavelength was 560 nm.

17. Characterizations

Hydrodynamic size and zeta potential of the NPs dispersed in water were measured by using a Zetasizer Nano ZS (Malvern Instruments) at room temperature. Catalytic activity of NPs and cascade reactions coupled with oxidases were studied by using a spectrophotometer (UV-3600 Plus, Shimadzu). A microplate reader (SpectraMax M2e, Molecular Device) was employed to study the detection performance of the NPs and to measure the fluorescent spectra of the NPs. A transmission electron microscope (TEM, Tecnai F20 microscope, FEI) was used to image NPs at an acceleration voltage of 200 kV. Morphology of NPs was also studied by using a scanning electron microscope (SEM, Zeiss Ultra 55 microscope). X-ray diffraction (XRD) patterns of particles were collected on a D8 ADVANCE diffractometer equipped with Cu radiation (λ = 1.54056 Å) at a scanning rate of 3–5 °/min . X-ray photoelectron spectroscopy (XPS) was performed on a PHI 5000 VersaProbe. The thermogravimetry (TG) analysis was performed on a TG 209 F3 (Netzsch) with a temperature ramp rate of 10 °C/min. N_2 isotherms of MOF nanozymes were obtained from an automatic volumetric gas adsorption analyser (ASAP 2460, Micromeritics).

Supplementary Figures

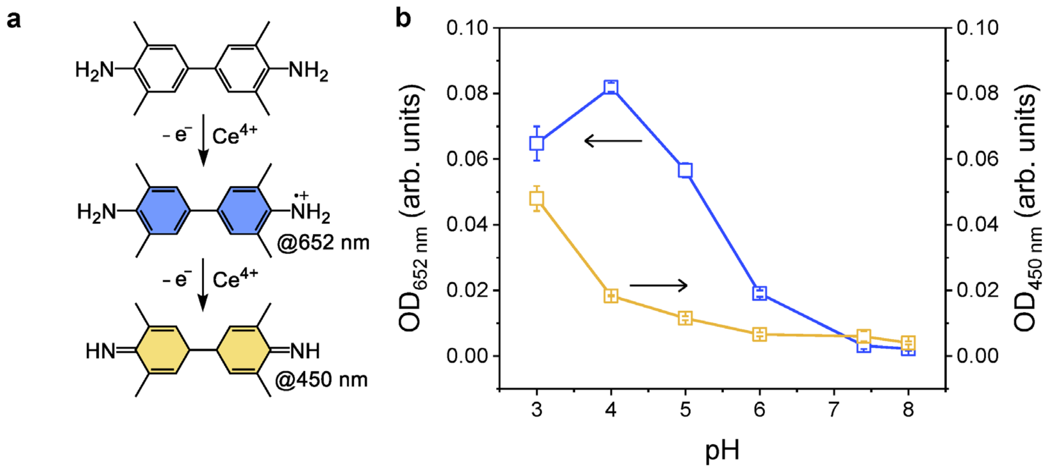

Supplementary Fig. 1. Charge transfer system containing TMB and Ce^{4+} ions at different pHs. (a) Schematic illustration of one-electron oxidation and two-electron oxidation of TMB, and its two oxidised TMB species exhibiting different characteristic absorption peaks. (b) Optical density at 652 nm ($OD_{652\ nm}$) and optical density at 450 nm ($OD_{450\ nm}$) of buffer solutions containing 50 μM TMB and 50 μM Ce^{4+} ions after 10-min reaction at different pHs. Data in (b) are expressed as mean values ± SEM, $n = 3$.

To prove the higher oxidation probability of TMB at lower pHs, we investigated a charge transfer system containing TMB and Ce^{4+} ions. TMB could be oxidised by Ce^{4+} ions, and we studied the influence of pH on this oxidation process. It is known that TMB undergoes both one-electron and two-electron oxidation processes[10], with the corresponding oxidised TMB possessing characteristic absorption peaks at 652 nm and 450 nm, respectively (Supplementary Fig. 1a). Hence, we monitored the absorption signals at both 652 nm and 450 nm in the presence of TMB and Ce^{4+} at different pHs. As shown in Supplementary Fig. 1b, when the pH decreased from 8.0 to 4.0, the signal at 652 nm increased. This result indicated that the formation of one-electron oxidised TMB increased with decrease of pH. It could be reasoned that TMB is prone to oxidation under acidic conditions. It was noted that the production of one-electron oxidised TMB decreased when the pH decreased from 4.0 to 3.0. In the meantime, the signal at 450 nm increased obviously, which proved that two-electron oxidised TMB was highly produced at pH 3.0. This result further confirmed the high oxidation probability of TMB at acidic pHs.

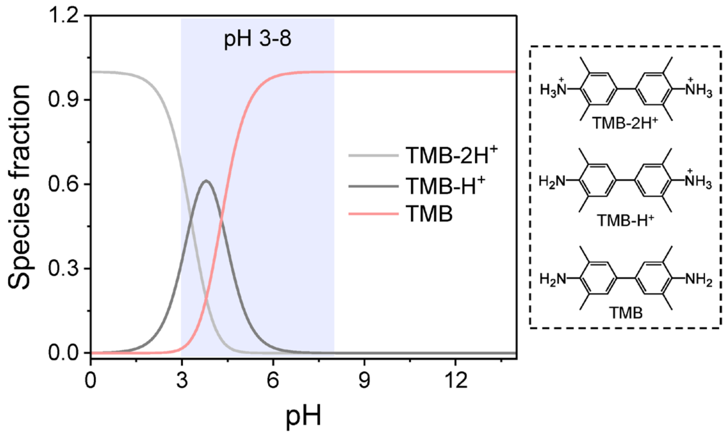

Supplementary Fig. 2. Bjerrum plot of TMB and its protonated species.

We speculated that the protonation of TMB increased its reducibility or oxidation probability. TMB is a typical benzidine compound, which possesses two pK_a values of around 3.3 and 4.3[11]. Hence, we depicted the Bjerrum plot of TMB and its protonated species (Supplementary Fig. 2). One TMB molecule adsorbed one proton or two protons, forming TMB-H^+ or TMB-$2H^+$, respectively. It is shown that TMB-$2H^+$ dominates at pH 3.0, while few TMB exists. When the pH increases to around 4.5, TMB-$2H^+$ essentially disappears and the fractions of TMB-H^+ and TMB are equivalent. With a further increase of pH to around 6.0, TMB-H^+ nearly disappears and the fraction of TMB is close to 1.0. When the pH increases to 7.4, TMB is the only species. In short, the amount of protonated TMB species decreases as pH increases. The Bjerrum plot corresponds quite well to the pH-dependent results about TMB oxidation by Ce^{4+} and TMB peroxidation by PCN-222-Fe NPs.

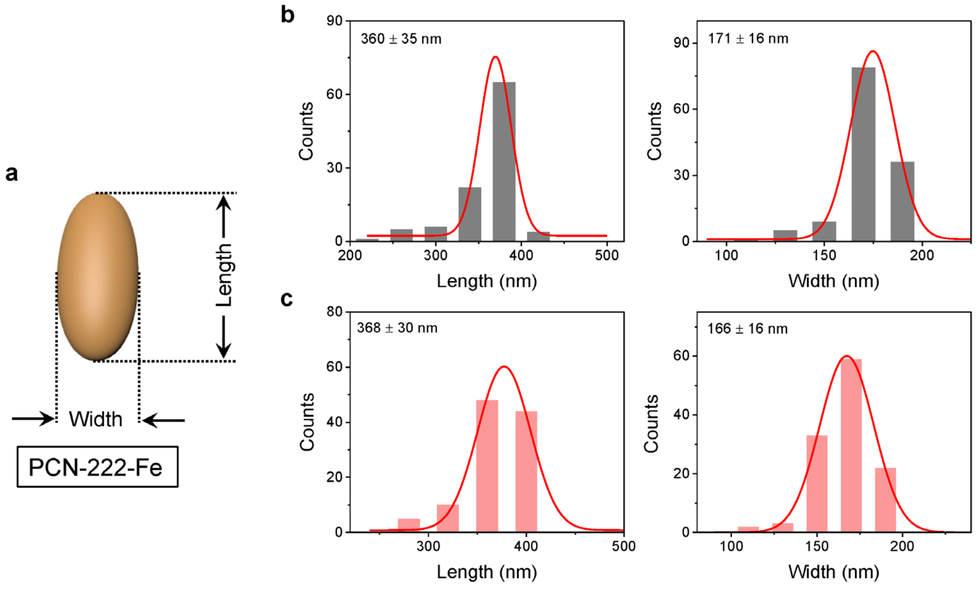

Supplementary Fig. 3. Length and width distribution of PCN-222-Fe NPs and PCN-222-Fe@PAA NPs. (a) Scheme of length and width of a PCN-222-Fe NP. Length and width distribution of (b) PCN-222-Fe NPs and (c) PCN-222-Fe@PAA NPs.

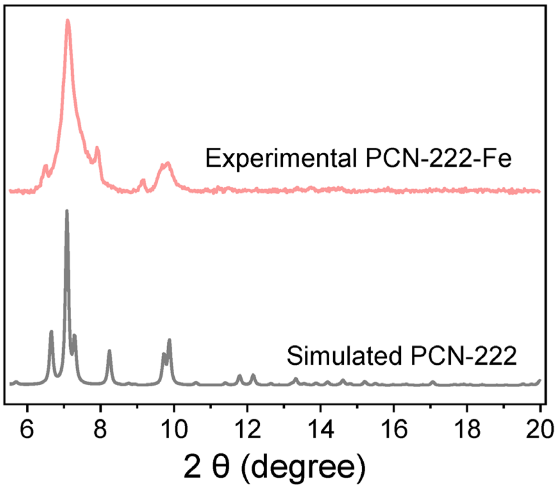

Supplementary Fig. 4. XRD patterns of PCN-222-Fe NPs and simulated PCN-222.

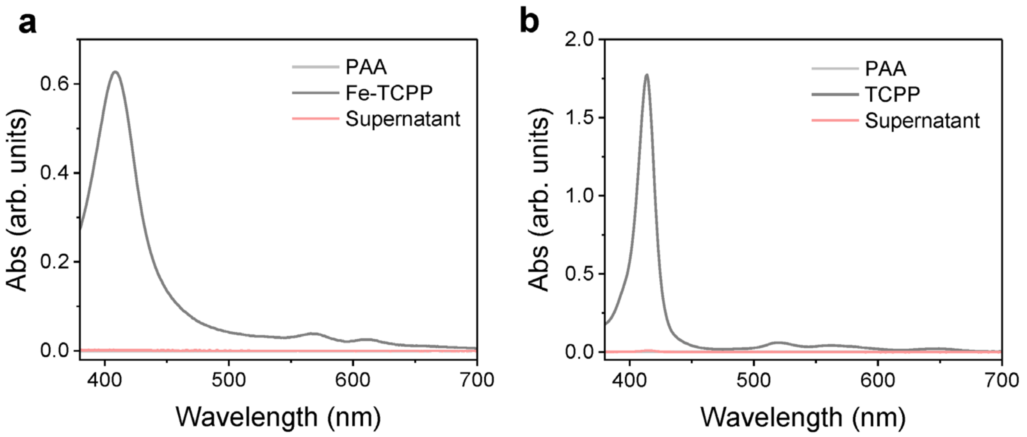

Supplementary Fig. 5. Influence of PAA incubation on linkers leaching in PCN-222-Fe NPs and PCN-222 NPs. (a) UV–vis spectra of 0.25 mg/mL PAA, 5 μg/mL Fe-TCPP, and the supernatant of 0.25 mg/mL PCN-222-Fe NPs after incubation with 0.5 mg/mL PAA. (b) UV–vis spectra of 0.25 mg/mL PAA, 5 μg/mL TCPP, and the supernatant of 0.25 mg/mL PCN-222 NPs after incubation with 0.5 mg/mL PAA. PAA, Fe-TCPP, and TCPP were dissolved in 100 mM phosphate buffer at pH 7.0. The supernatant was added into an equal volume of 200 mM phosphate buffer at pH 7.0 before UV–vis measurements.

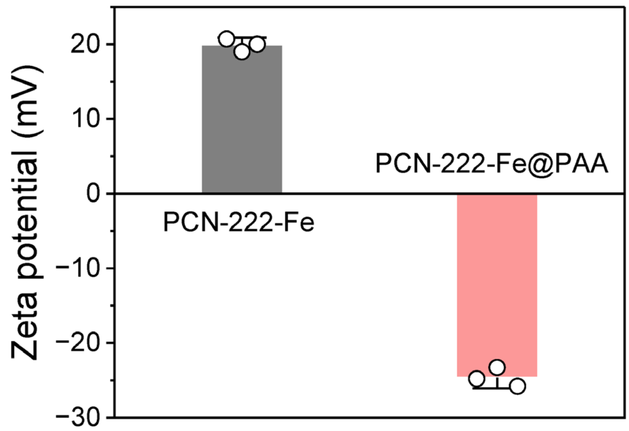

Supplementary Fig. 6. Zeta potentials of PCN-222-Fe NPs and PCN-222-Fe@PAA NPs. Data are expressed as mean values ± SEM, *n* = 3.

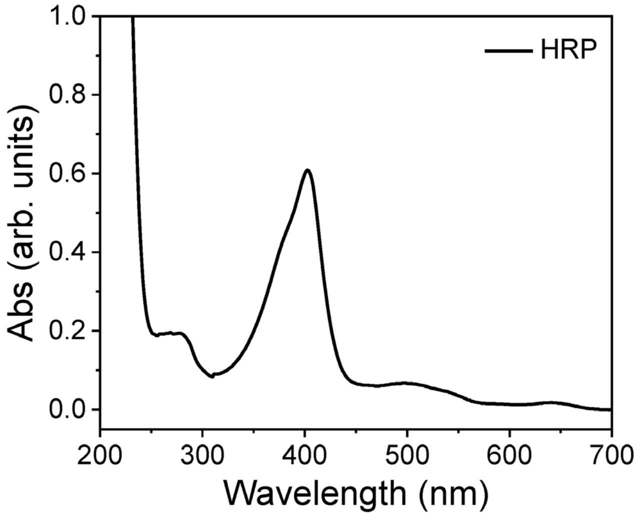

Supplementary Fig. 7. Measurement of the molar concentration of HRP solution.

The absorbance of 0.25 mg/mL HRP solution at 403 nm is 0.608, giving a molar concentration of 6.08 μM with an absorbance coefficient of 1.0×10^5 M^{-1}•cm^{-1}.

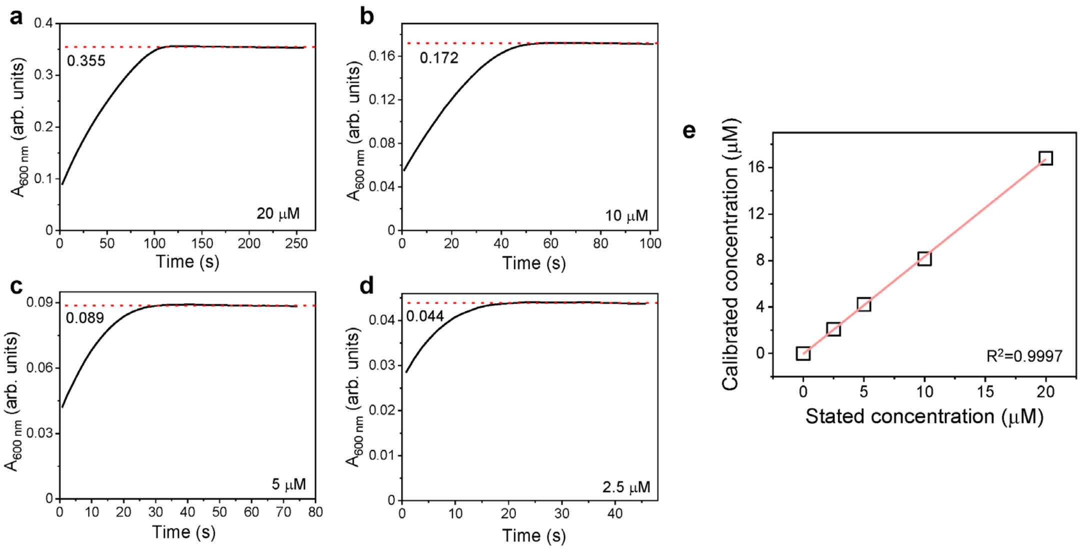

Supplementary Fig. 8. H_2O_2 calibration. (a-d) Time evolution of absorbance at 600 nm ($A_{600\ nm}$) for monitoring the oxidation of 0.2 mM ABTS in the presence of 20 nM HRP and H_2O_2 with different concentrations. H_2O_2 concentrations listed inside graphs are the stated concentrations. (e) Calibration curve of H_2O_2.

We calibrated the commercial H_2O_2 (30 wt%) by using HRP and its substrate, 2,2'-azino-bis(3-ethylbenzothiazoline-6-sulfonic acid) (ABTS). ABTS can be catalytically oxidised into cationic radicals, $ABTS^{+\bullet}$, with a coefficient of $1.1 \times 10^4\ M^{-1}\bullet cm^{-1}$ at 600 nm. The concentration of the commercial H_2O_2 (30 wt%) was stated to be 9.9 M, which was diluted to several concentrations ranging from 2.5 μM to 20 μM in the reaction solutions. The absorbance changes of ABTS solutions at 600 nm reached a plateau very soon with the addition of HRP and several diluted H_2O_2, indicating the complete consumption of H_2O_2. The corresponding absorbance values at the plateau were recorded. The actual concentrations can be calculated by using the recorded absorbance values. The plot of the calibrated H_2O_2 concentrations against the stated concentrations was fitted linearly, giving a slope of 0.838. Thus, the concentration of the H_2O_2 stocking solution was 8.3 M (multiplying 9.9 M by 0.838).

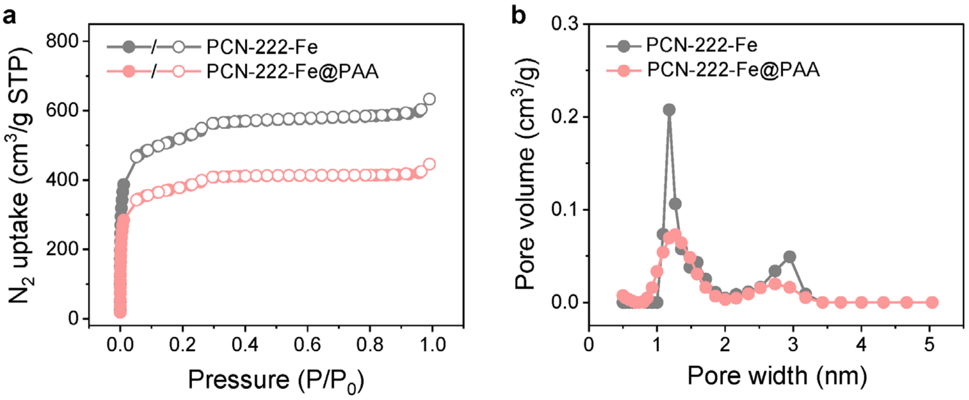

Supplementary Fig. 9. (a) N_2 isotherms and (b) pore size distributions of PCN-222-Fe NPs and PCN-222-Fe@PAA NPs.

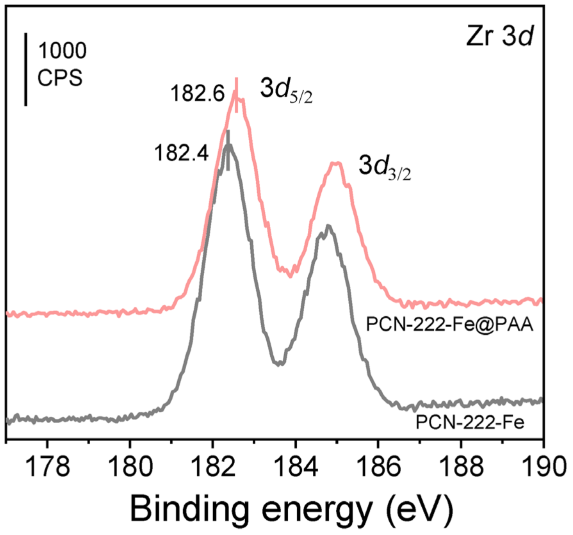

Supplementary Fig. 10. Zr 3*d* XPS spectra of PCN-222-Fe NPs and PCN-222-Fe@PAA NPs.

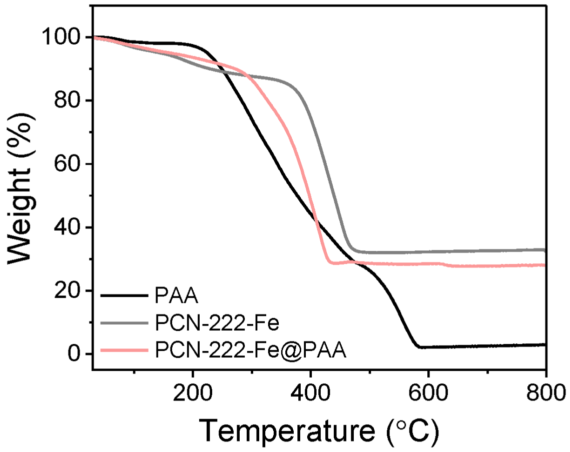

Supplementary Fig. 11. TG curves of PAA, PCN-222-Fe NPs, and PCN-222-Fe@PAA NPs.

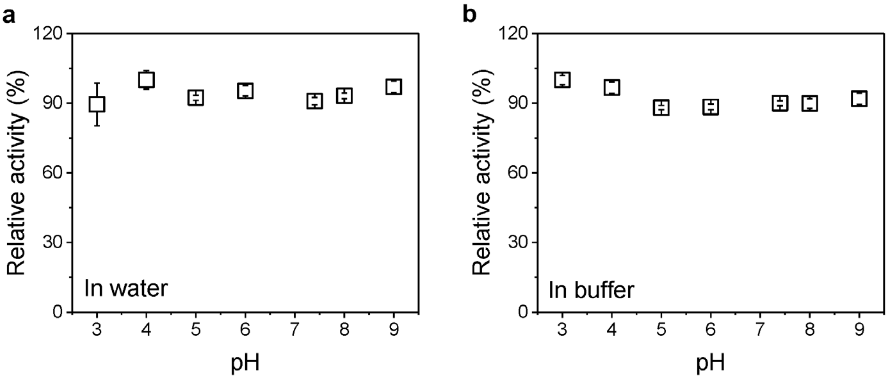

Supplementary Fig. 12. pH-dependent operational stability of PCN-222-Fe NPs. Relative activity of PCN-222-Fe NPs after 1-h incubation at room temperature in (a) water, and (b) buffer solutions at different pHs. The pH of water in panel a was adjusted by HCl or NaOH. Data in (a and b) are expressed as mean values ± SEM, $n = 3$.

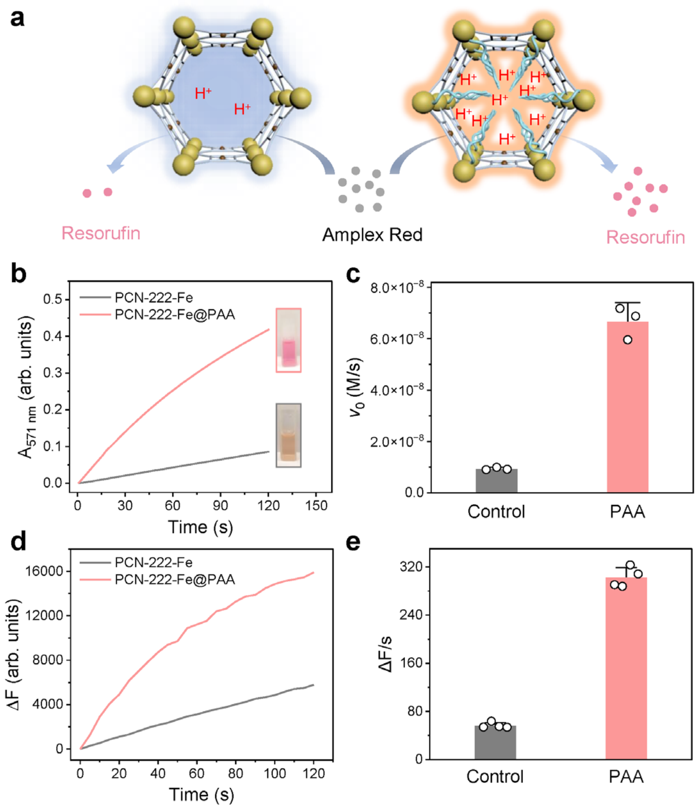

Supplementary Fig. 13. Improved activity for a fluorescent substrate, Amplex Red, at physiological pH. (a) Schematic illustration of activity improvement of PCN-222-Fe NPs for Amplex Red with the production of Resorufin through PAA modification. (b) Time evolution of absorbance at 571 nm ($A_{571\ nm}$) for monitoring the peroxidation of 0.1 mM Amplex Red catalysed by 5 µg/mL PCN-222-Fe NPs or PCN-222-Fe@PAA NPs. (c) The initial velocities corresponding to panel b. (d) Time evolution of the fluorescent emission intensity change (ΔF) at 585 nm with λ_{ex} = 560 nm under the same catalytic condition used in panel b. (e) The initial velocities corresponding to panel d. Data in (c) are expressed as mean values ± SEM, n = 3. Data in (e) are expressed as mean values ± SEM, n = 4.

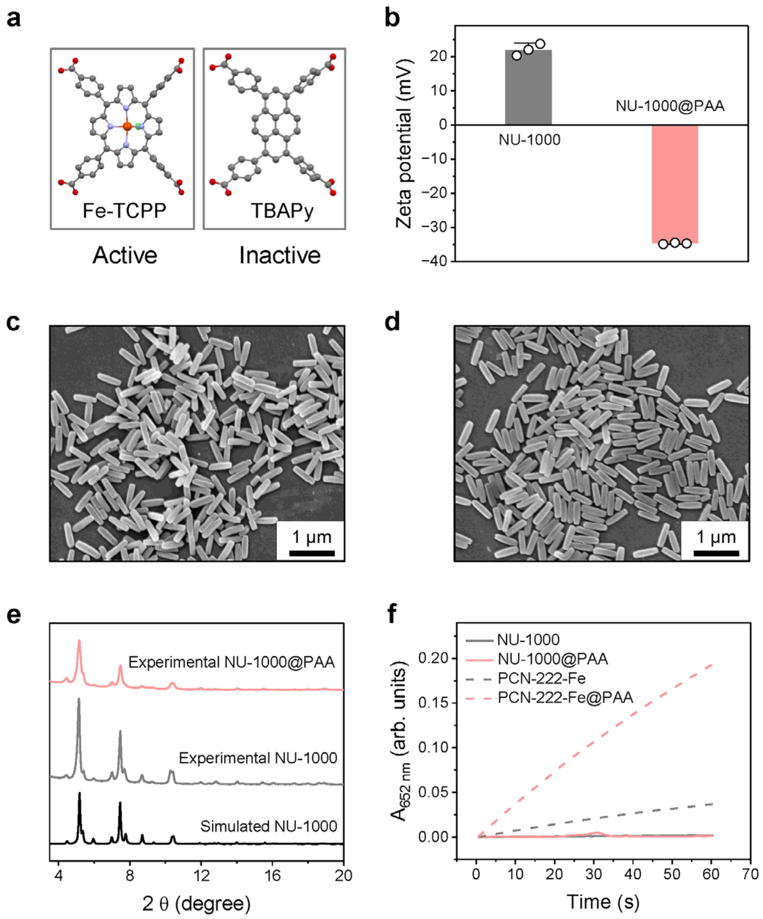

Supplementary Fig. 14. Organic linkers in PCN-222-Fe NPs serving as catalytically active sites. (a) Molecular structures of Fe-TCPP and TBAPy based on ball-and-stick model. Red: O, Grey: C, Purple: N, Brown: Fe, Green: Cl. (b) Zeta potentials of NU-1000 NPs and NU-1000@PAA NPs. Representative SEM images of (c) NU-1000 NPs and (d) NU-1000@PAA NPs. (e) XRD patterns of NU-1000 NPs, NU-1000@PAA NPs, and simulated NU-1000 NPs. (f) Time evolution of absorbance at 652 nm ($A_{652\ nm}$) for monitoring the peroxidase-mimicking catalytic activities of 5 μg/mL NU-1000 NPs and NU-1000@PAA NPs at R.T., under the condition of 200 mM Tris buffer (pH 7.4) containing 0.2 mM TMB and 0.2 mM H_2O_2. Activity measurements of PCN-222-Fe NPs and PCN-222-Fe@PAA NPs were shown for comparison. Data in (b) are expressed as mean values ± SEM, $n = 3$.

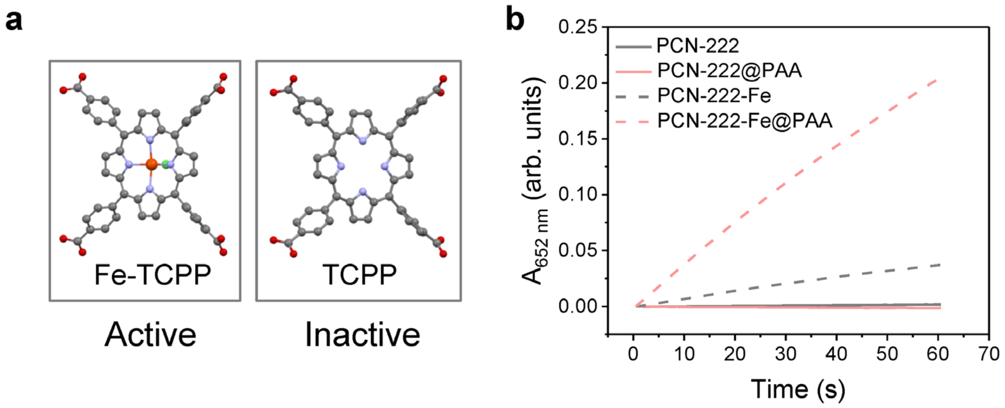

Supplementary Fig. 15. Iron porphyrin linkers serving as catalytically active sites. (a) Molecular structures of Fe-TCPP and TCPP based on ball-and-stick model. Red: O, Grey: C, Purple: N, Brown: Fe, Green: Cl. (b) Time evolution of $A_{652\ nm}$ for monitoring the peroxidase-mimicking catalytic activities of 5 µg/mL PCN-222 NPs and PCN-222@PAA NPs at R.T., under the condition of 200 mM Tris buffer (pH 7.4) containing 0.2 mM TMB and 0.2 mM H_2O_2. Activity measurements of PCN-222-Fe NPs and PCN-222-Fe@PAA NPs were shown for comparison.

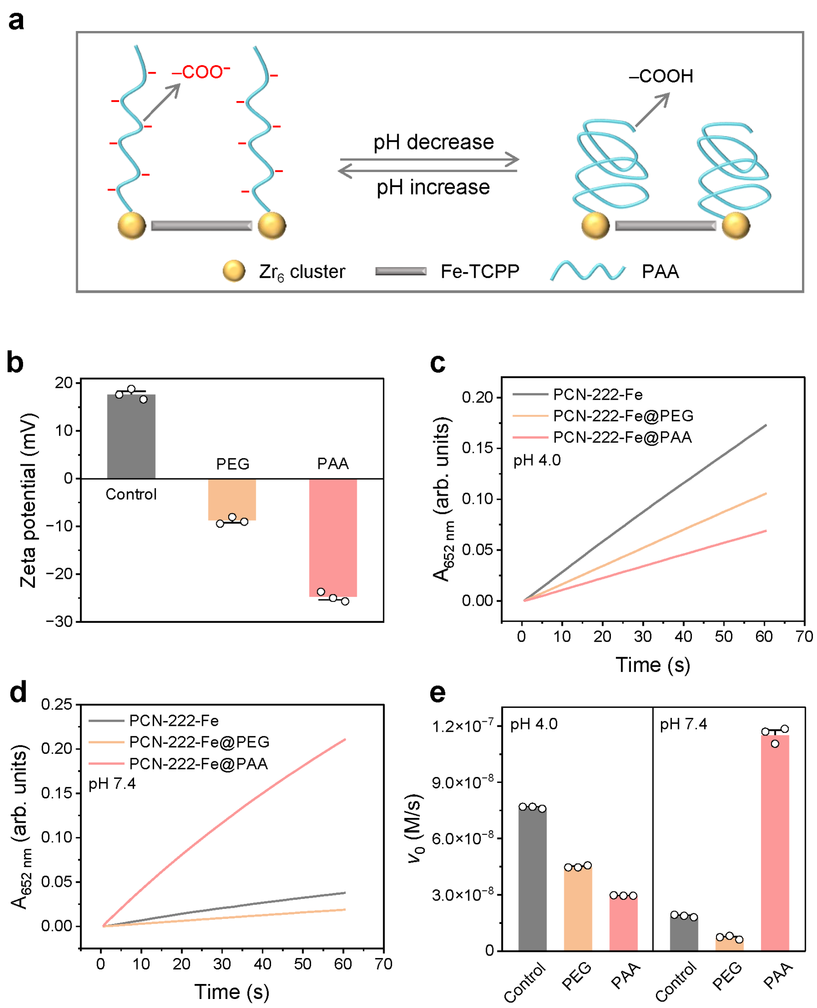

Supplementary Fig. 16. Activity decrease of PCN-222-Fe NPs induced by PAA confinement under acidic condition. (a) Schematic illustration of conformational change of PAA chain induced by pH change. (b) Zeta potentials of PCN-222-Fe NPs before and after modification of PEG and PAA. PEG used here is COOH-PEG-COOH with a M_w of 2 kDa. Time evolution of absorbance at 652 nm ($A_{652\ nm}$) for monitoring the peroxidase-mimicking catalytic activities PCN-222 NPs, PCN-222@PEG NPs, and PCN-222-Fe@PAA at (c) pH 4.0 and (d) pH 7.4. (e) Initial velocities corresponding to panels c–d. Data in (b and e) are expressed as mean values ± SEM, *n* = 3.

PAA is pH-responsive. It shrinks with decrease of pH and expands with increase of pH, arising from protonation and deprotonation of carboxyl groups in PAA chain.[12-13] It is proposed that PAA shrinks under acidic condition due to protonation of carboxyl groups, which obstructs the catalytically active sites leading to an activity decrease (Supplementary Fig. 16a). To illustrate this, we employed PEG with an identical M_w to PAA, and PEG used here possesses two end carboxyl groups which can coordinate with Zr_6 clusters in PCN-222-Fe NPs. Supplementary Fig. 16b shows that PEG modification

leads to a slightly negative zeta potential, which proves the successful modification of PEG. Unlike PAA, PEG is not pH-responsive, and it is shown that PEG modification leads to activity decrease of PCN-222-Fe NPs at both pHs 4.0 and 7.4, which can be ascribed to the obstruction of catalytically active sites (*i.e.*, Fe-TCPP) (Supplementary Fig. 16c–e). Hence, it is supposed that PAA chains obstruct the catalytically active sites under acidic condition leading to activity decrease of PCN-222-Fe NPs. When the pH of reaction solution increased to 7.4, the expanded PAA chains might partially block the catalytically active sites. Nevertheless, the local pH decrease enabled through deprotonation of PAA chains could play a dominant role in activity improvement of PCN-222-Fe NPs. Combined, an activity improvement rather than a decrease was observed under neutral conditions.

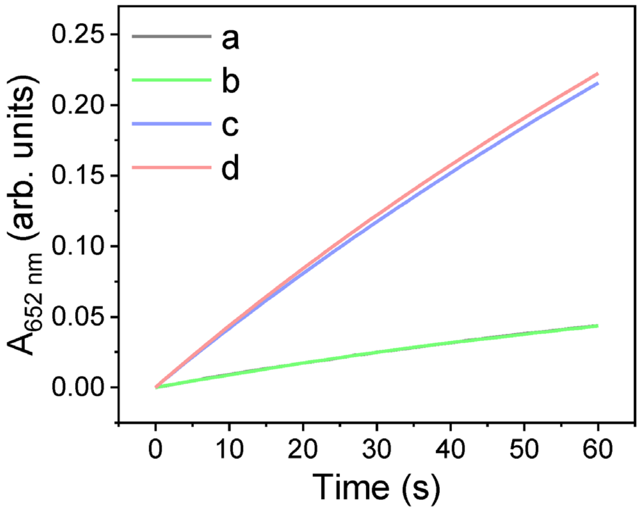

Supplementary Fig. 17. PAA incubation achieves the improved peroxidase-like activity of PCN-222-Fe NPs. Time evolution of absorbance at 652 nm ($A_{652\ nm}$) for monitoring the oxidation of 0.2 mM TMB in the presence of 0.2 mM H_2O_2 and PCN-222 NPs (curve a), PCN-222 NPs and PAA (curve b), PCN-222 NPs after 30-min incubation with PAA (curve c), and PCN-222 NPs after 30-min incubation with PAA, centrifugation and redispersion in water (curve d). PCN-222 NPs = 5 μg/mL, pH = 7.4.

The PCN-222-Fe@PAA NPs employed for the increased peroxidase-like activity measurement (Fig. 2e–f) were obtained by incubating PCN-222-Fe NPs with PAA for 30 min without removal of free PAA. Therefore, we further investigated the activity of PCN-222-Fe@PAA NPs before and after the removal of free PAA. For both cases, the increased peroxidase-like activities were observed (Supplementary Fig. 17. curves c and d). These results further confirm that the increased peroxidase-like activity resulted from the confined PAA.

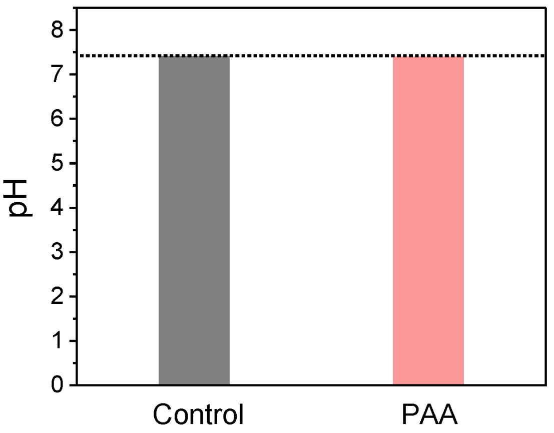

Supplementary Fig. 18. Effect of PAA on the bulk pH of Tris buffer. Tris buffer = 0.2 M, PAA = 10 μg/mL.

We studied the effect of PAA on the bulk pH of Tris buffer solution. It is demonstrated that 10 μg/mL PAA, the amount used for the activity modulation of PCN-222-Fe NPs, does not change the pH of Tris buffer solution. This result rules out the potential role of PAA-induced change of bulk solution.

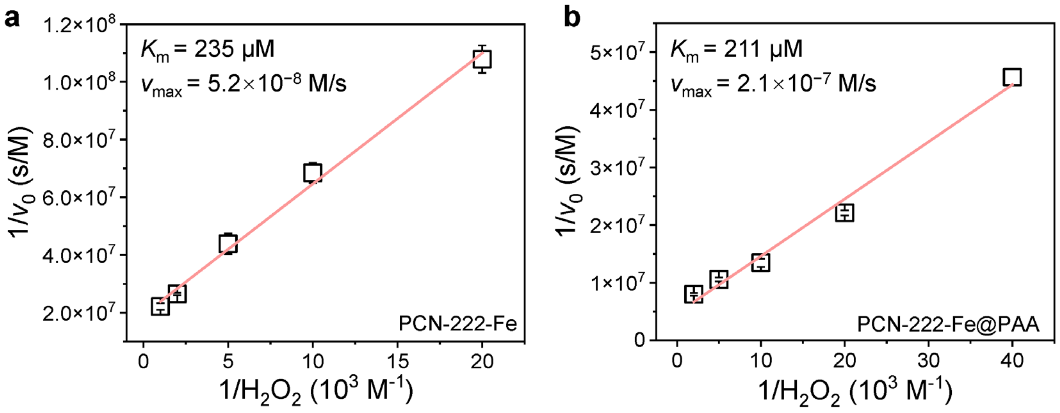

Supplementary Fig. 19. **Lineweaver–Burk plots of reciprocal velocities against reciprocal H_2O_2 concentrations.** (a) PCN-222-Fe NPs and (b) PCN-222-Fe@PAA NPs. TMB = 0.2 mM, PCN-222-Fe NPs = 5 μg/mL, pH = 7.4. Data in (a and b) are expressed as mean values ± SEM, n = 3. v_{max} stands for the maximal velocity, and K_m stands for the Michaelis constant.

The maximal velocity of PCN-222-Fe@PAA NPs (~2.1×10^{-7} M/s) is about 4-fold that of PCN-222-Fe NPs (~5.2×10^{-8} M/s) and the K_m value for H_2O_2 decreases from 235 μM to 211 μM after PAA modification. While the decreased K_m value indicates that PCN-222-Fe@PAA NPs possess improved affinity towards H_2O_2 compared with PCN-222-Fe NPs, such a small decrease of K_m value after PAA modification is not significant enough to boost the observed activity.

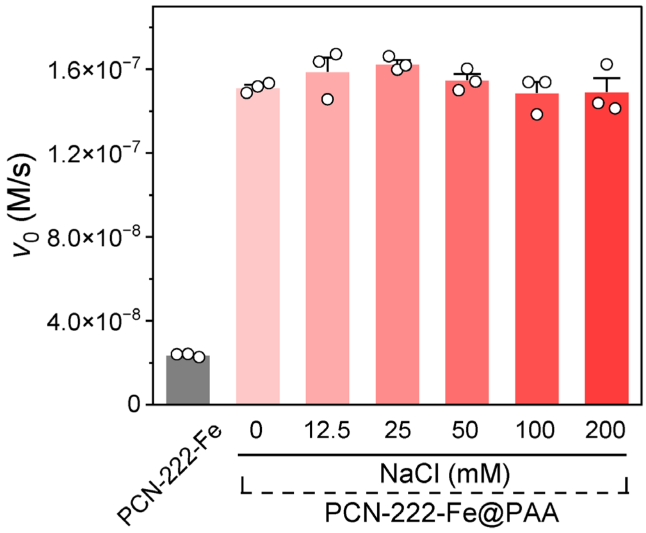

Supplementary Fig. 20. Influence of NaCl concentration on the peroxidase-like activity of PCN-222-Fe@PAA NPs. Data are expressed as mean values ± SEM, n = 3. v_0 stands for initial velocity.

To exclude the possible electrostatic interaction between PCN-222-Fe@PAA NPs and substrate TMB, NaCl with different concentrations ranging from 12.5 mM to 200 mM was employed to study its effect on the peroxidase-like activity of PCN-222-Fe@PAA NPs. It is shown that the introduction of NaCl barely influences the activity of PCN-222-Fe@PAA NPs for TMB, which indicates that the improved activity by PAA confinement does not come from electrostatic force between the NPs and TMB. This is mainly because TMB is not positively charged at pH 7.4, although PCN-222-Fe@PAA NPs exhibit a negative zeta potential (Supplementary Fig. 6).

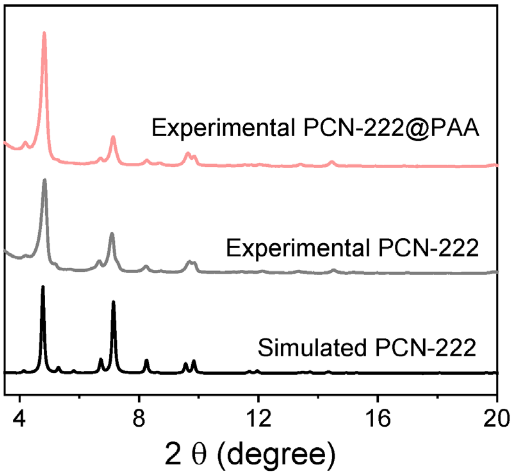

Supplementary Fig. 21. XRD patterns of PCN-222 NPs before and after PAA modification, and simulated PCN-222.

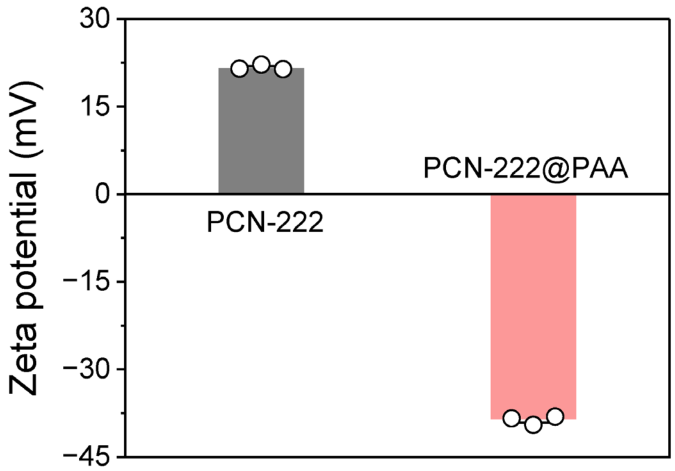

Supplementary Fig. 22. Zeta potentials of PCN-222 NPs and PCN-222@PAA NPs. Data are expressed as mean values ± SEM, $n = 3$.

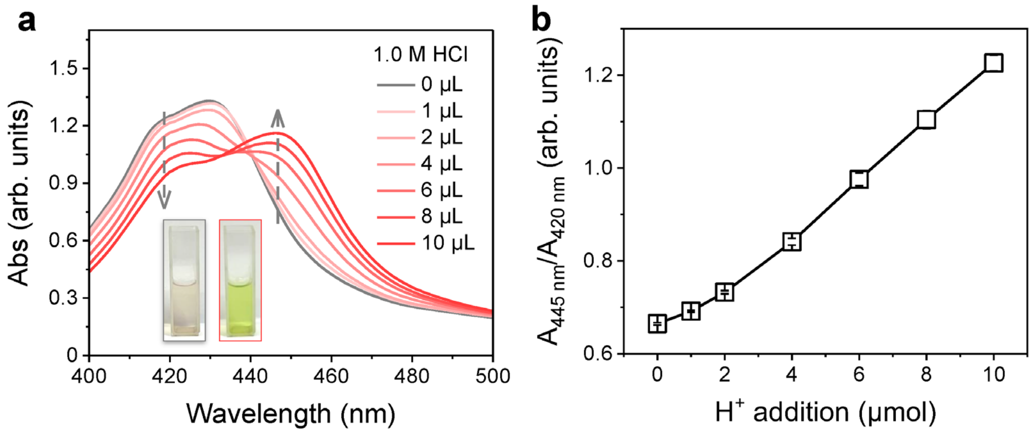

Supplementary Fig. 23. Influence of protons addition on the light absorption of PCN-222 NPs. (a) Absorption spectra of 2 mL of 10 μg/mL PCN-222 NPs with the addition of 1.0 M HCl. The inset photos show the colour change of PCN-222 NPs before and after the addition of 10 μL of 1.0 M HCl. (b) Plot of the ratio of absorbance at 445 nm to absorbance at 420 nm ($A_{445\ nm}/A_{420\ nm}$) versus amounts of protons added corresponding to panel a. Data in (b) are expressed as mean values ± SEM, *n* = 3.

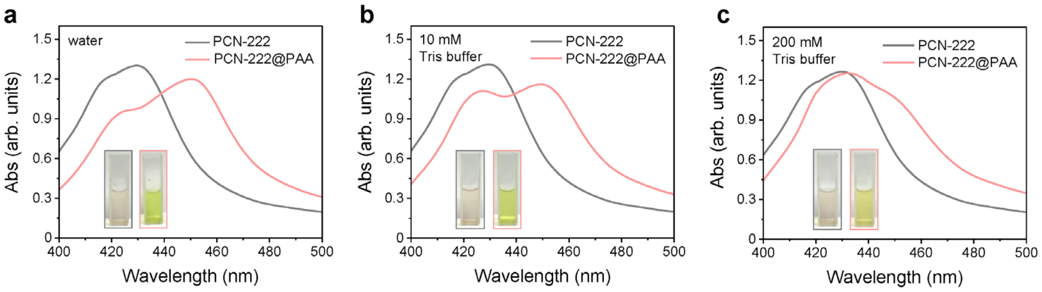

Supplementary Fig. 24. Absorption spectra of PCN-222 NPs and PCN-222@PAA NPs. Absorption spectra of 2 mL of 10 μg/mL PCN-222 NPs and PCN-222@PAA NPs in (a) water, (b) 10 mM Tris buffer, and (c) 200 mM Tris buffer, pH = 7.4. The inset photo shows the colours of PCN-222 NPs and PCN-222@PAA NPs.

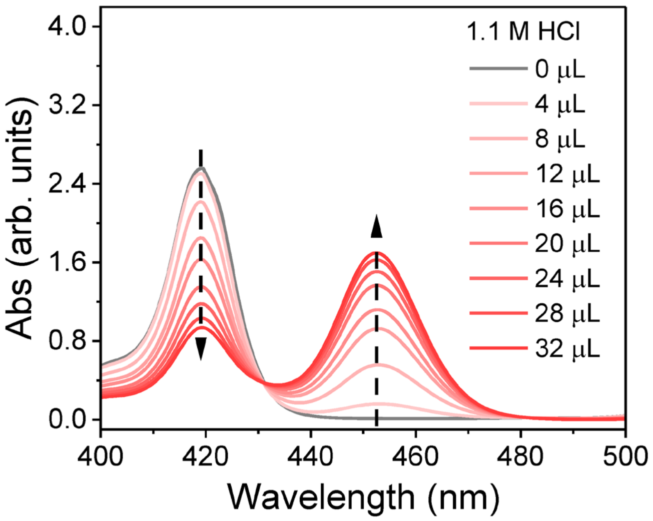

Supplementary Fig. 25. Absorption spectra of TCPP DMF solution with addition of protons.

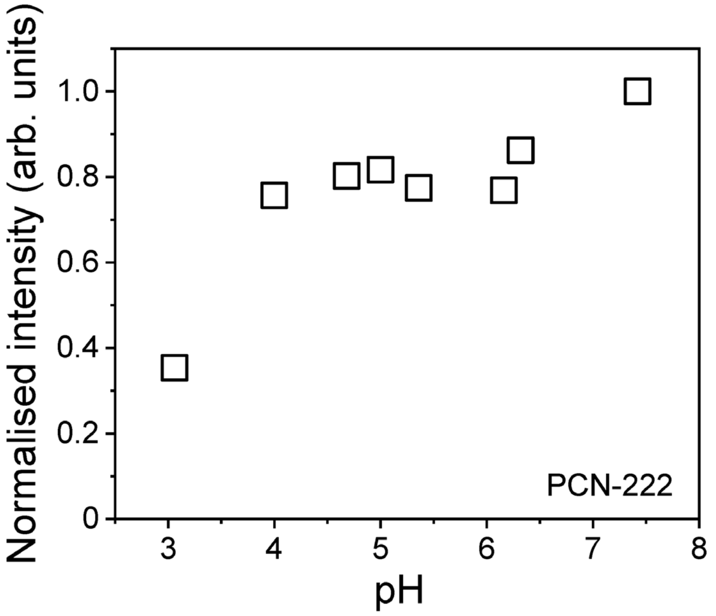

Supplementary Fig. 26. Normalised fluorescence intensity of PCN-222 NPs at 660 nm at different pHs. PCN-222 NPs = 20 μg/mL, λ_{ex} = 420 nm.

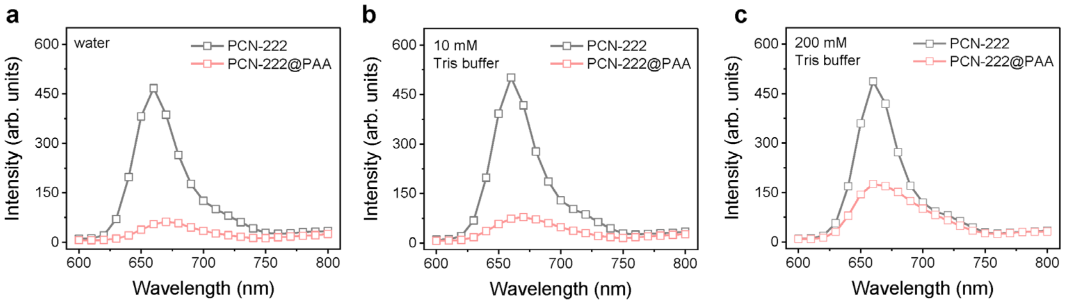

Supplementary Fig. 27. Fluorescence emission spectra of PCN-222 NPs and PCN-222@PAA NPs. Fluorescence emission spectra of 20 μg/mL PCN-222 NPs and PCN-222@PAA NPs in (a) water, (b) 10 mM Tris buffer, and (c) 200 mM Tris buffer, pH = 7.4. λ_{ex} = 420 nm.

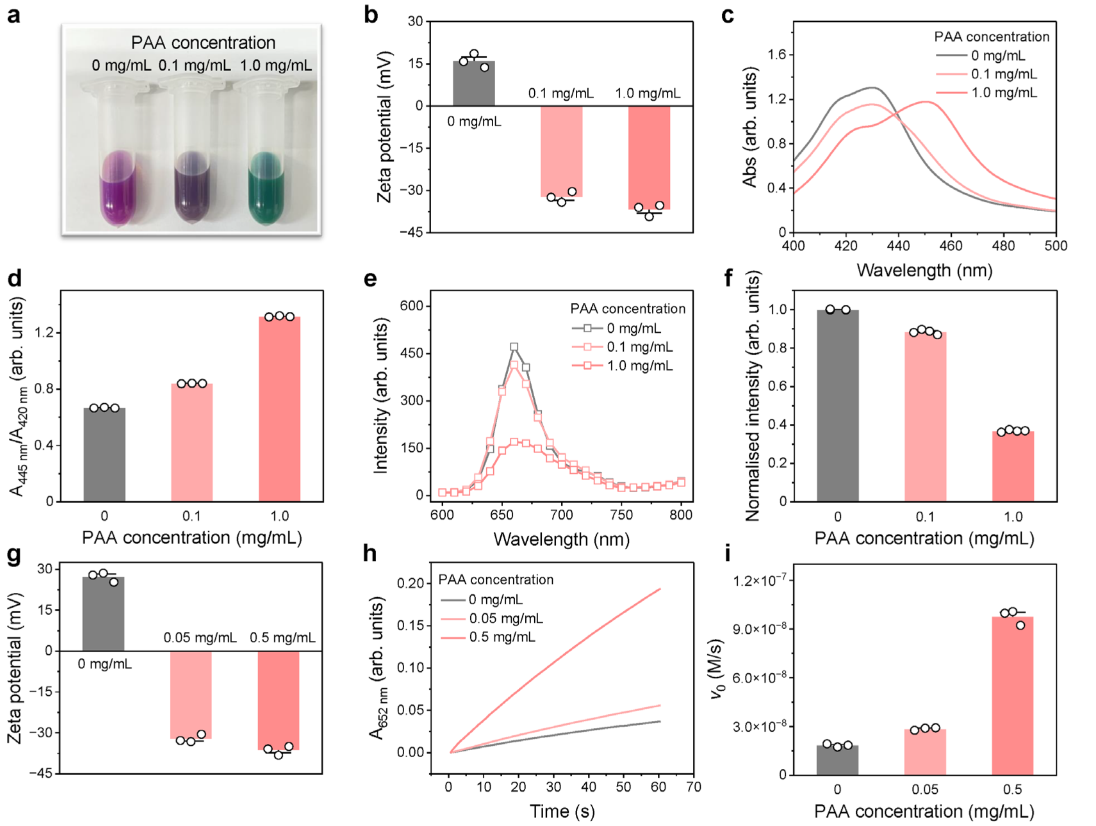

Supplementary Fig. 28. Influence of PAA concentration on the microenvironmental pH decrease. (a) Photos showing the colour changes of 0.5 mg/mL PCN-222 NPs after incubation with 0.1 mg/mL PAA and 1.0 mg/mL PAA. (b) Zeta potentials of 0.25 mg/mL PCN-222-Fe NPs before and after modification with 0.05 mg/mL PAA and 0.5 mg/mL PAA. (c) Absorption spectra of 2 mL of 10 µg/mL PCN-222 NPs before and after modification with PAA. (d) Ratio of absorbance at 445 nm to absorbance at 420 nm ($A_{445\ nm}/A_{420\ nm}$) corresponding to panel c. (e) Fluorescence emission spectra of 20 µg/mL PCN-222 NPs before and after modification with PAA in 200 mM Tris buffer, pH = 7.4. λ_{ex} = 420 nm. (f) Normalised fluorescence intensities at 660 nm corresponding to panel e. (g) Zeta potentials of 0.25 mg/mL PCN-222-Fe NPs before and after modification with 0.05 mg/mL PAA and 0.5 mg/mL PAA. (h) Time evolution of absorbance at 652 nm ($A_{652\ nm}$) for monitoring the peroxidase-like catalytic activities of 5 µg/mL PCN-222-Fe NPs before and after modification with PAA at R.T., under the condition of 200 mM Tris buffer (pH 7.4) containing 0.2 mM TMB and 0.2 mM H_2O_2. (i) Initial velocities (v_0) corresponding to panel h. Data in (d, g, and i) are expressed as mean values ± SEM, n = 3. Data in (f) are expressed as mean values ± SEM, n = 4.

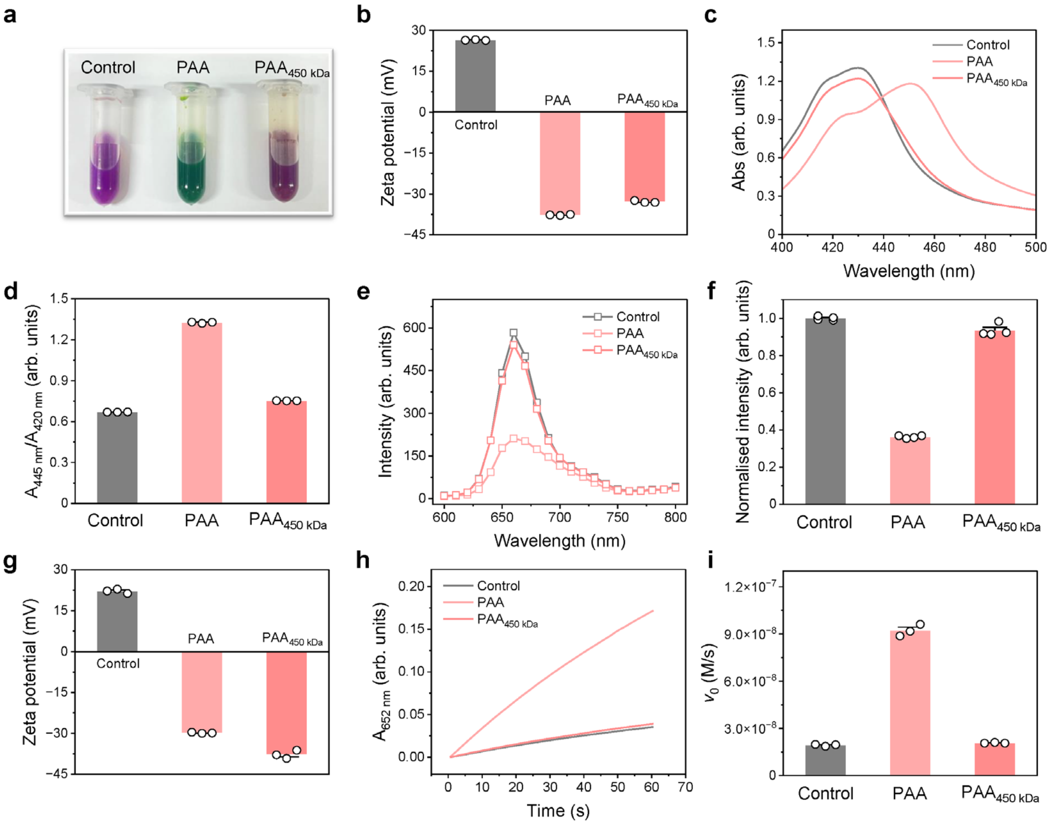

Supplementary Fig. 29. Influence of PAA molecular weight on the microenvironmental pH decrease. (a) Photos showing the colour changes of 0.5 mg/mL PCN-222 NPs after incubation with 1.0 mg/mL PAA and 1.0 mg/mL $PAA_{450\ kDa}$. (b) Zeta potentials of PCN-222 NPs, PCN-222@PAA NPs, and PCN-222@$PAA_{450\ kDa}$ NPs. (c) Absorption spectra of 2 mL of 10 µg/mL PCN-222 NPs before and after modification with PAA and $PAA_{450\ kDa}$. (d) Ratio of absorbance at 445 nm to absorbance at 420 nm ($A_{445\ nm}/A_{420\ nm}$) corresponding to panel c. (e) Fluorescence emission spectra of 20 µg/mL PCN-222 NPs before and after modification with PAA and $PAA_{450\ kDa}$ in 200 mM Tris buffer, pH = 7.4. λ_{ex} = 420 nm. (f) Normalised fluorescence intensities at 660 nm corresponding to panel e. (g) Zeta potentials of PCN-222-Fe NPs before and after modification with PAA and $PAA_{450\ kDa}$. (h) Time evolution of absorbance at 652 nm ($A_{652\ nm}$) for monitoring the peroxidase-like catalytic activities of 5 µg/mL PCN-222-Fe NPs before and after modification with PAA and $PAA_{450\ kDa}$ at R.T., under the condition of 200 mM Tris buffer (pH 7.4) containing 0.2 mM TMB and 0.2 mM H_2O_2. (i) Initial velocities (v_0) corresponding to panel h. Data in (d, g, and i) are expressed as mean values ± SEM, n = 3. Data in (f) are expressed as mean values ± SEM, n = 4.

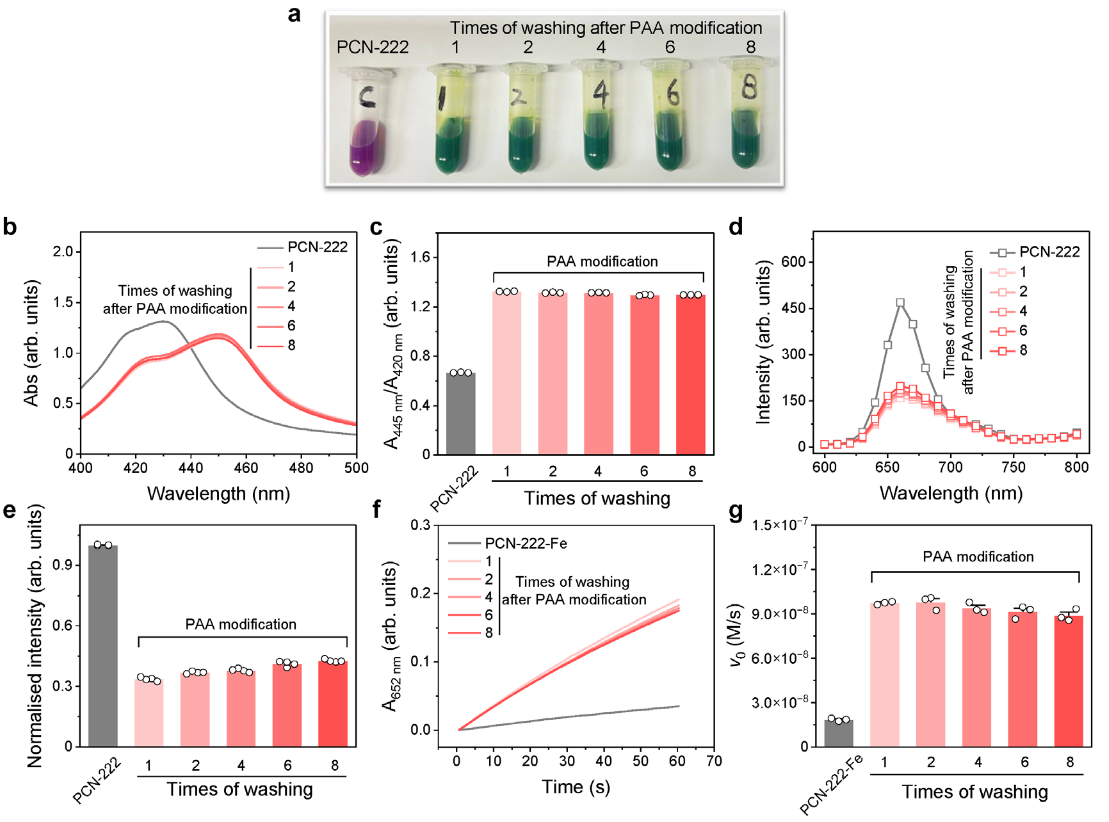

Supplementary Fig. 30. Microenvironmental pH decrease enabled by PAA confinement after thorough washing. (a) Photos showing that 0.5 mg/mL PCN-222@PAA NPs remain green after thorough washing. (b) Absorption spectra of 2 mL of 10 μg/mL PCN-222 NPs and PCN-222@PAA NPs after thorough washing. (c) Plot of the ratio of absorbance at 445 nm to absorbance at 420 nm ($A_{445\ nm}/A_{420\ nm}$) versus times of washing corresponding to panel b. (d) Fluorescence emission spectra of 20 μg/mL PCN-222 NPs and PCN-222@PAA NPs after thorough washing in 200 mM Tris buffer, pH = 7.4. λ_{ex} = 420 nm. (e) Normalised fluorescence intensities at 660 nm corresponding to panel d. (f) Time evolution of $A_{652\ nm}$ for monitoring the peroxidase-mimicking catalytic activities of 5 μg/mL PCN-222-Fe NPs or PCN-222-Fe@PAA NPs after thorough washing at R.T., under the condition of 200 mM Tris buffer (pH 7.4) containing 0.2 mM TMB and 0.2 mM H_2O_2. (g) Initial velocities (v_0) corresponding to panel f. Data in (c and g) are expressed as mean values ± SEM, n = 3. Data in (e) are expressed as mean values ± SEM, n = 4.

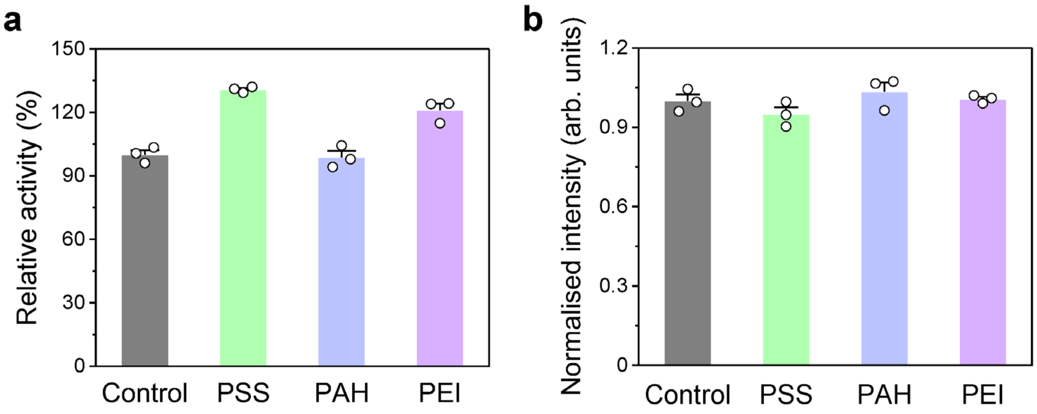

Supplementary Fig. 31. Effect of other polymers on the activity of PCN-222-Fe NPs. (a) Relative activity of PCN-222-Fe NPs after incubation with several polymers including PAA, PSS, PAH, and PEI. (b) Normalised fluorescence intensity of PCN-222 NPs at 660 nm in water after incubation of several polymers corresponding to panel a. λ_{ex} = 420 nm. Data in (a and b) are expressed as mean values ± SEM, n = 3.

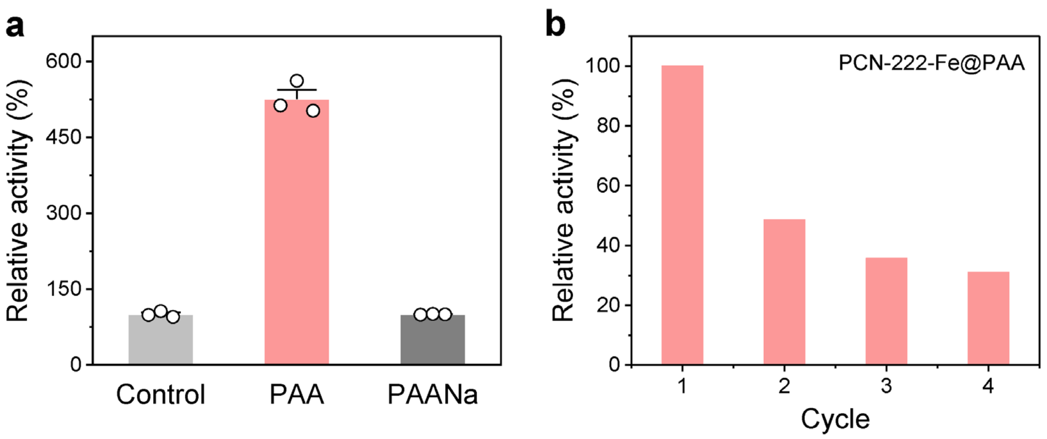

Supplementary Fig. 32. Deprotonated PAA does not increase the peroxidase-like activity of PCN-222-Fe NPs. (a) Influence of modification with PAA and deprotonated PAA (*i.e.*, PAANa) on the peroxidase-like activity of PCN-222-Fe NPs. (b) Influence of the catalysis cycle on the peroxidase-like activity of PCN-222-Fe@PAA NPs. Data in (a) are expressed as mean values ± SEM, $n = 3$.

Deprotonated PAA, termed as PAANa, is not able to improve the peroxidase-mimicking activity of PCN-222-Fe NPs as PAA (Supplementary Fig. 32a). The result indicated that protons donation by PAA is essential to activity enhancement of PCN-222-Fe NPs. Considering that peroxidation of reducing substrates consumed protons, the activity of PCN-222-Fe@PAA NPs decays during catalysis cycles (Supplementary Fig. 32b).

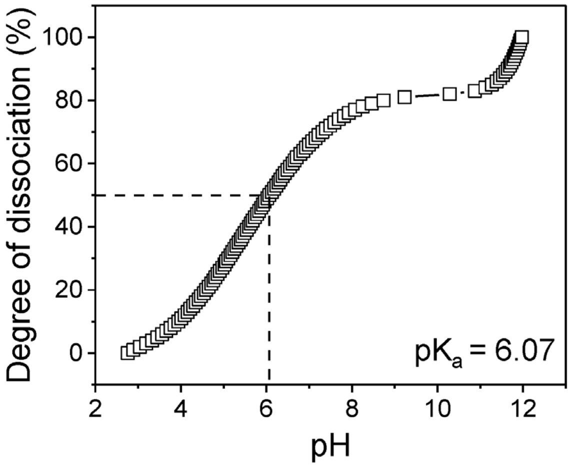

Supplementary Fig. 33. Titration curve of PAA. pK_a stands for dissociation constant.

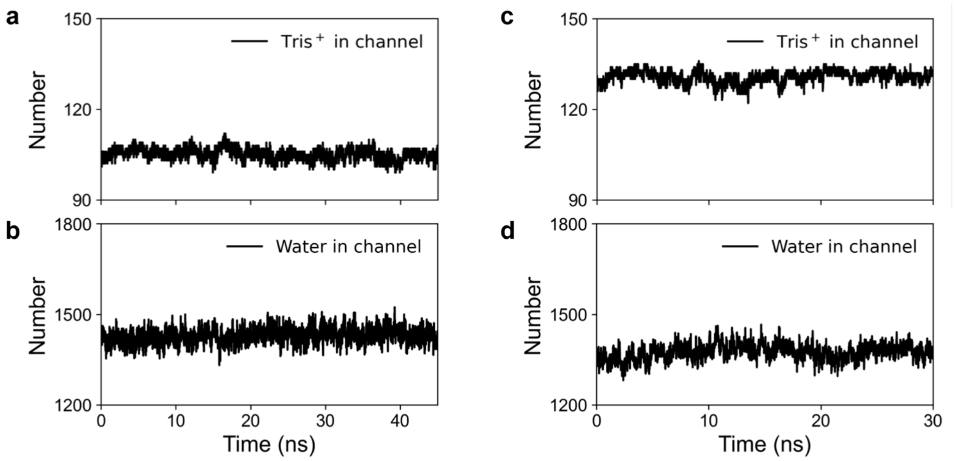

Supplementary Fig. 34. Equilibrium of the number of Tris and water in a PAA-modified channel in MD simulations. The equilibrium of the number of (a) Tris and (b) water in a PAA-modified channel for the 22/5 system. The information for the 27/0 system is shown in (c-d).

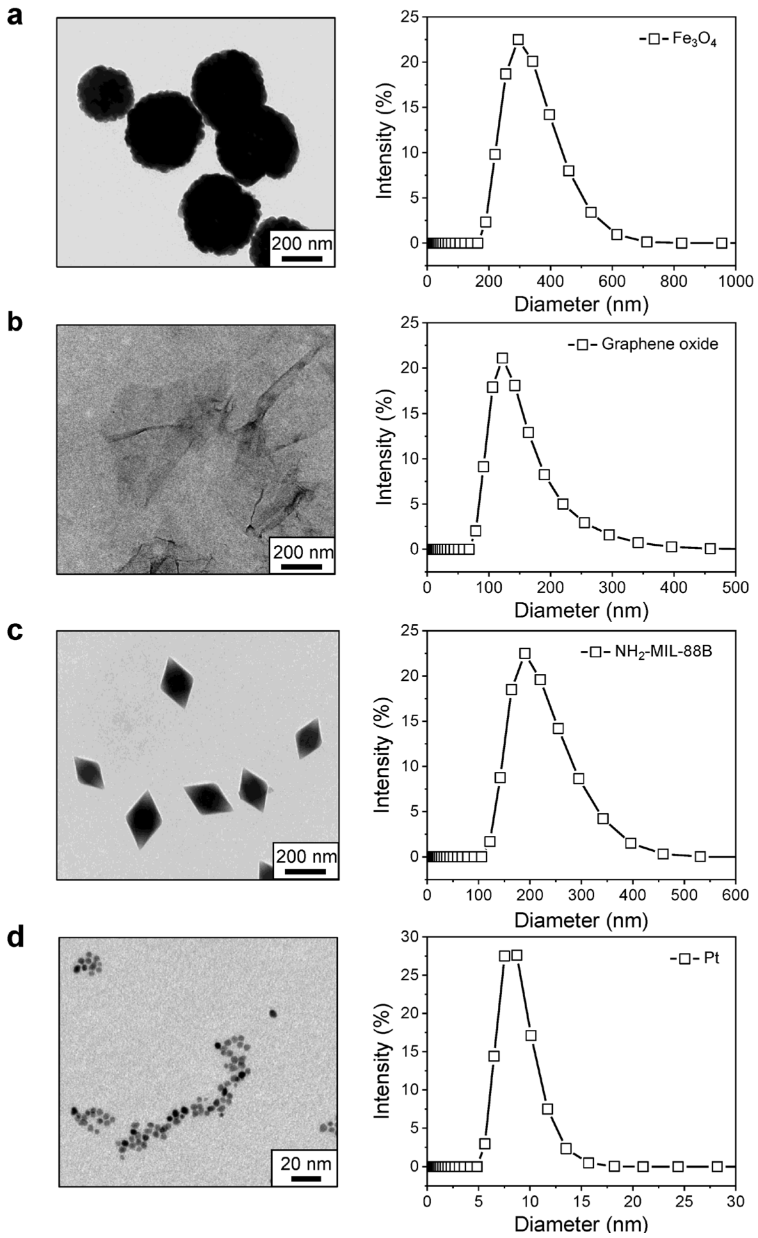

Supplementary Fig. 35. Representative TEM images and hydrodynamic size distributions of (a) Fe_3O_4 NPs, (b) graphene oxide, (c) NH_2-MIL-88B NPs, and (d) Pt NPs obtained from one batch of NPs.

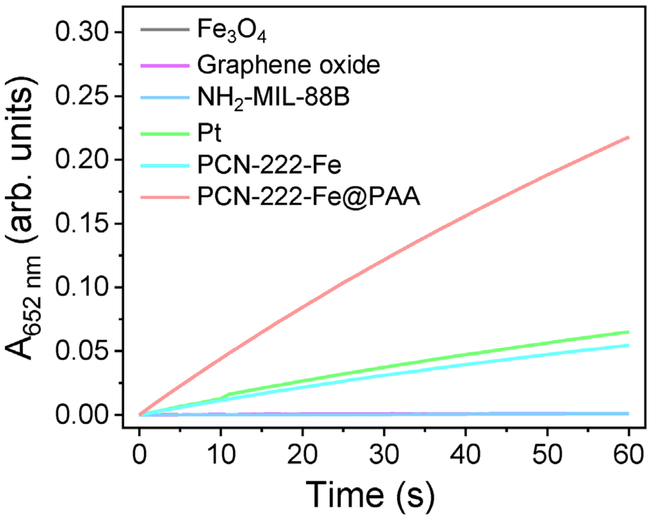

Supplementary Fig. 36. Activity comparison with other nanozymes. Time evolution of absorbance at 652 nm ($A_{652\ nm}$) for monitoring the oxidation of 0.2 mM TMB in the presence of 5 μg/mL nanozymes and 0.2 mM H_2O_2 at pH = 7.4.

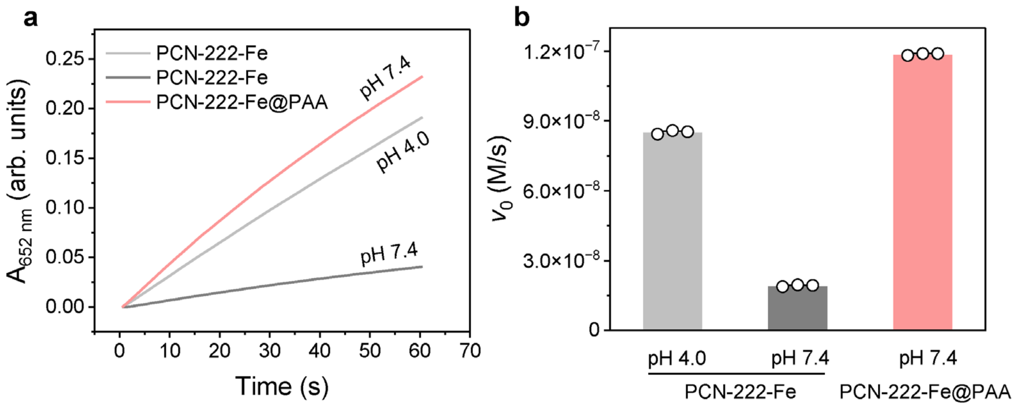

Supplementary Fig. 37. PAA modification increases the peroxidase-like activity of PCN-222-Fe NPs at pH 7.4. (a) Time evolution of absorbance at 652 nm ($A_{652\ nm}$) for monitoring the peroxidase-mimicking activity of PCN-222 NPs at pHs 4.0 and 7.4, and PCN-222-Fe@PAA NPs at pH 7.4. (b) Initial velocities (v_0) corresponding to panel a. Data in (b) are expressed as mean values ± SEM, n = 3.

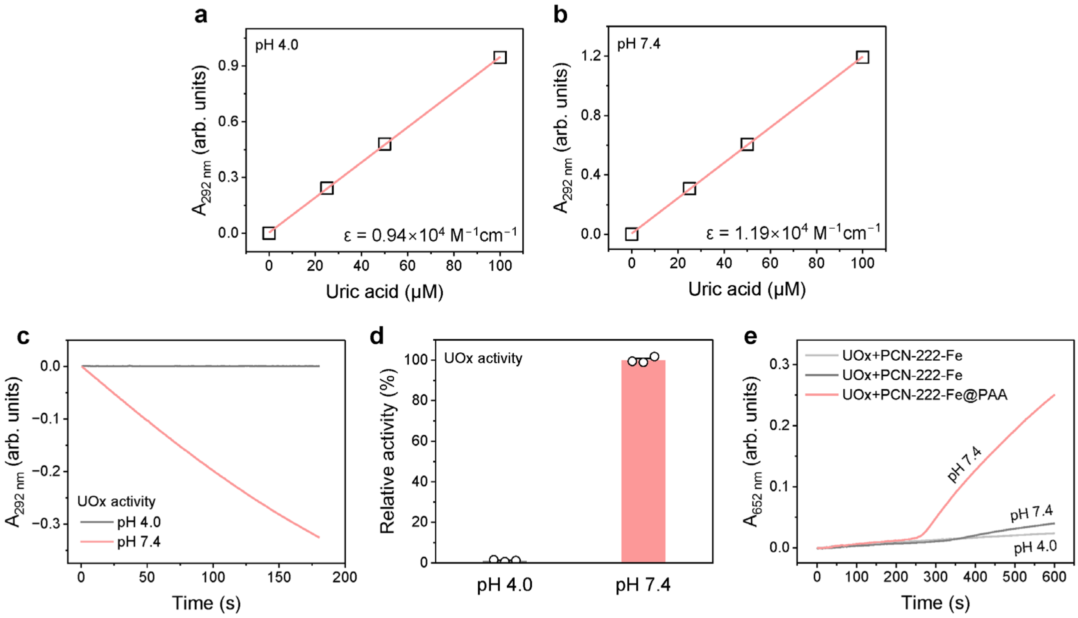

Supplementary Fig. 38. Activity measurement of urate oxidase (UOx) and one-pot oxidase/MOF nanozyme cascade reaction. Absorbance at 292 nm ($A_{292\ nm}$) versus uric acid concentration at (a) pH 4.0 and (b) pH 7.4. (c) Time evolution of $A_{292\ nm}$ for monitoring the activity of UOx at pHs 4.0 and 7.4. (d) Relative activity of UOx at pHs 4.0 and 7.4. (e) Time evolution of absorbance at 652 nm ($A_{652\ nm}$) for monitoring one-pot cascade reactions catalysed by UOx and coupled with PCN-222-Fe NPs at pHs 4.0 and 7.4, or PCN-222-Fe@PAA NPs at pH 7.4. Data in (d) are expressed as mean values ± SEM, $n = 3$.

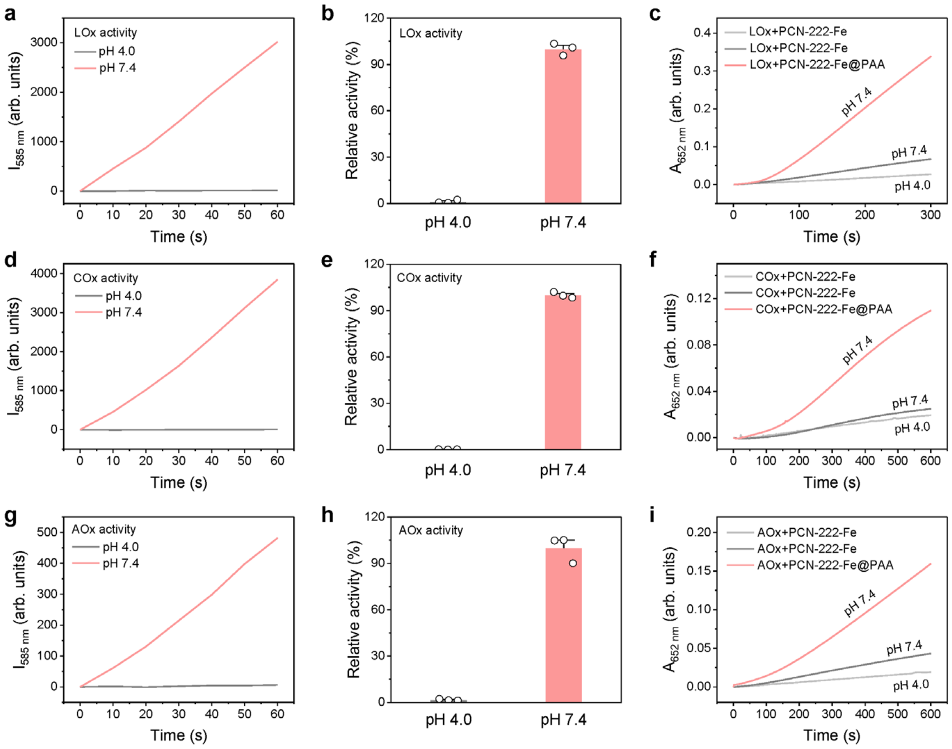

Supplementary Fig. 39. Activity measurement of four representative oxidases and one-pot oxidase/MOF nanozyme cascade reactions. Time evolution of fluorescence intensity at 585 nm ($I_{585\ nm}$) for monitoring the catalytic activity of (a) lactate oxidase (LOx), (d) choline oxidase (COx), and (g) alcohol oxidase (AOx) at pHs 4.0 and 7.4. Relative activity of (b) LOx, (e) COx, and (h) AOx corresponding to panels a, d, and g, respectively. Time evolution of absorbance at 652 nm ($A_{652\ nm}$) for monitoring one-pot cascade reactions catalysed by (c) LOx, (f) COx, and (i) AOx coupled with PCN-222-Fe NPs at pHs 4.0 and 7.4, and PCN-222-Fe@PAA NPs at pH 7.4. λ_{ex} = 560 nm. Data in (b, e, and h) are expressed as mean values ± SEM, *n* = 3.

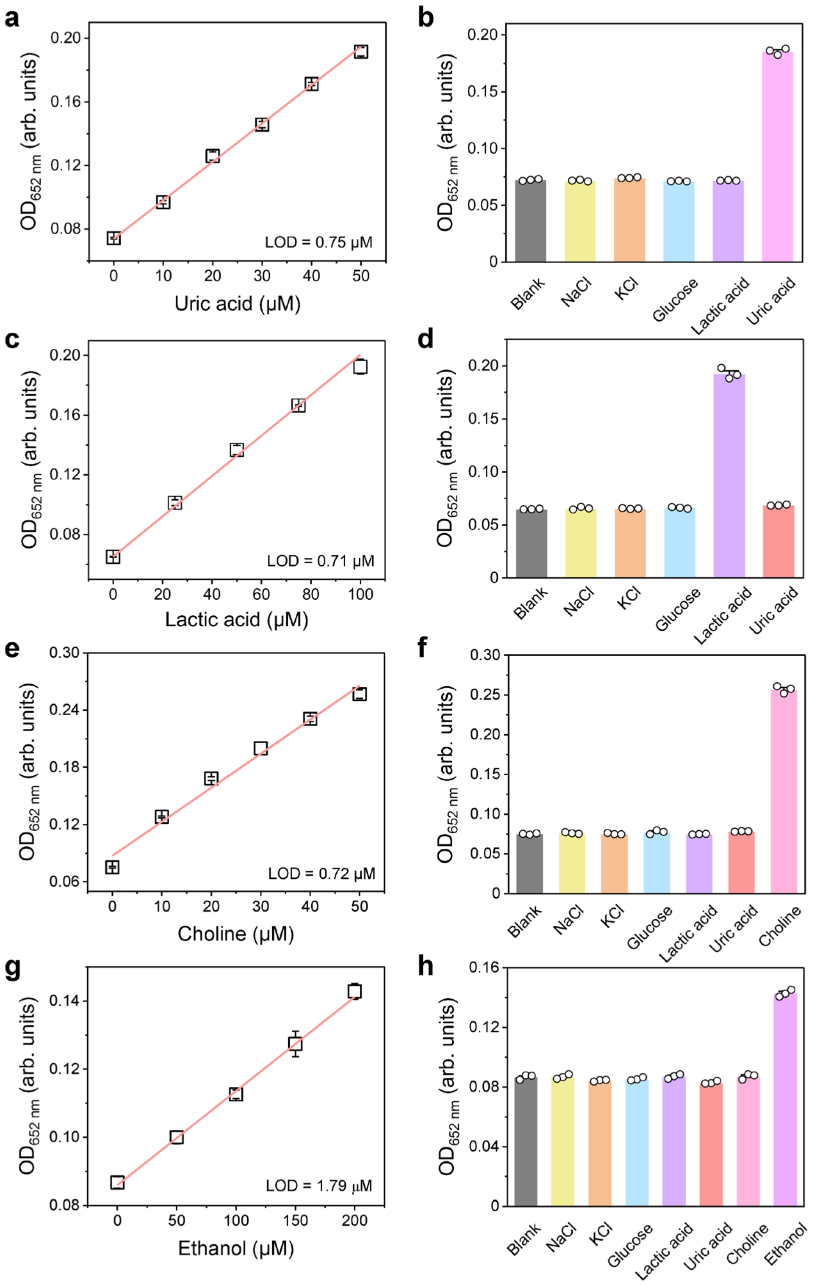

Supplementary Fig. 40. Biomolecules sensing enabled by oxidase/PCN-222-Fe@PAA cascade reactions. Detection of (a) uric acid, (c) lactic acid, (e) choline, and (g) ethanol enabled by cascade reactions catalyzed by UOx, LOx, COx, and AOx, respectively coupled with PCN-222-Fe@PAA NPs at pH 7.4. Detection selectivity towards (b) uric acid, (d) lactic acid, (f) choline, and (h) ethanol, and the interferences concentration was five times targets concentration. $OD_{652\ nm}$ stands for optical density at 652 nm. Data in (a–h) are expressed as mean values ± SEM, $n = 3$.

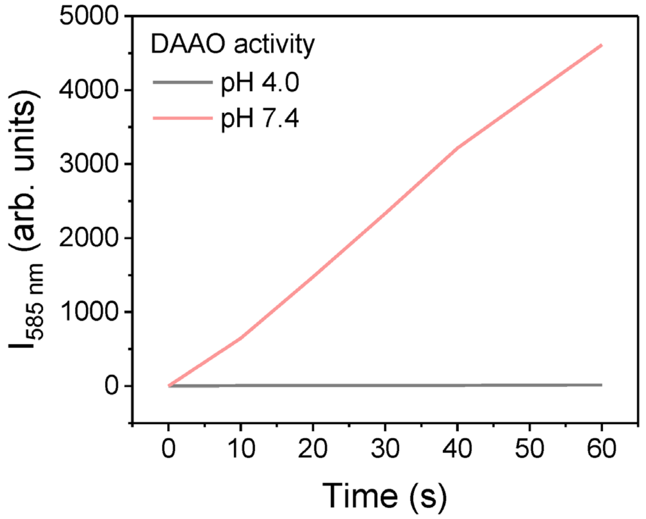

Supplementary Fig. 41. Activity measurement of D-amino acid oxidase (DAAO). Time evolution of fluorescence intensity at 585 nm ($I_{585\ nm}$) for monitoring the catalytic activity of DAAO at pHs 4.0 and 7.4.

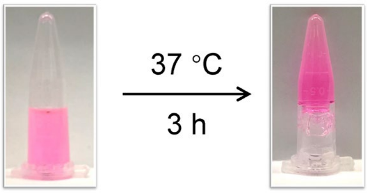

Supplementary Fig. 42. Gelation of PEI and PEGDE in ethanol.

The concentration of PEI and PEGDE was 10%, and 10 μg/mL phenol red was used for colouring the gel. The formation of gel in ethanol proves the reactivity between the primary amine groups of PEI and the epoxide groups of PEGDE.

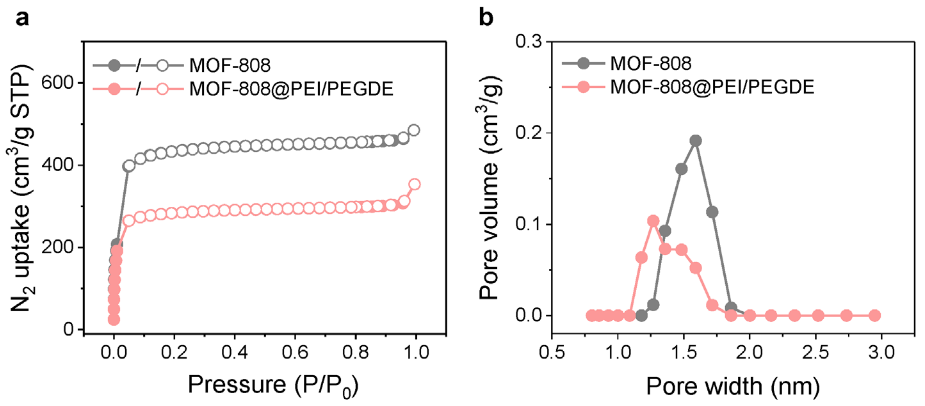

Supplementary Fig. 43. (a) N_2 isotherms and (b) pore size distributions of MOF-808 NPs and MOF-808@PEI/PEGDE NPs.

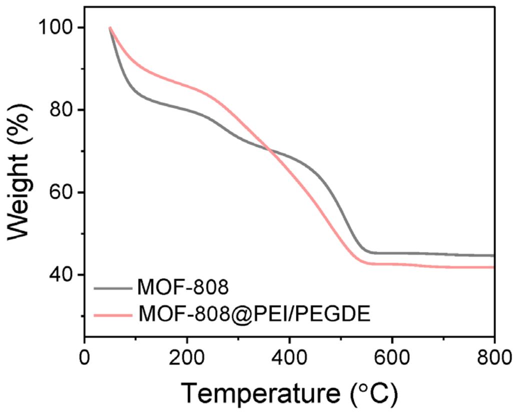

Supplementary Fig. 44. TG curves of MOF-808 NPs and MOF-808@PEI/PEGDE NPs.

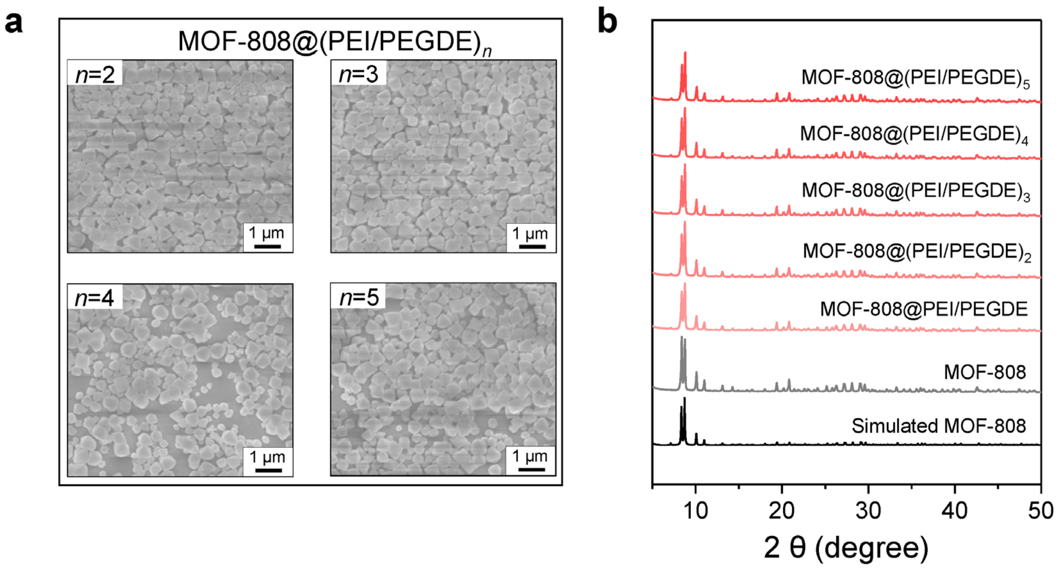

Supplementary Fig. 45. Representative SEM images and XRD patterns of MOF-808@(PEI/PEGDE)$_n$ NPs. n = 0, 1, 2, 3, 4, and 5. SEM images and XRD patterns were obtained from one batch of NPs.

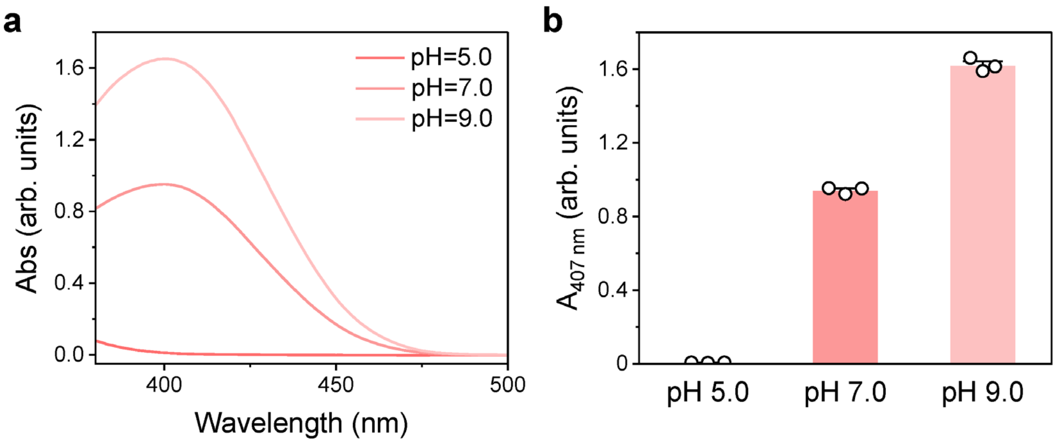

Supplementary Fig. 46. Influence of pH on the absorption of pNP. (a) UV–vis spectra of 0.1 mM pNP in NaAc/HAc buffer at pH 5.0, K_2HPO_4/KH_2PO_4 buffer at pH 7.0, and Tris/HCl buffer at pH 9.0. (b) Absorbance values at 407 nm ($A_{407\ nm}$) corresponding to panel a. The buffer concentration was 50 mM. Data in (b) are expressed as mean values ± SEM, *n* = 3.

Before measuring the hydrolytic activity of MOF nanozymes, we observed that the product of the hydrolysis of pNPP, pNP, exhibited significant differences in the absorbance at 407 nm with varied pHs. Hence, we compared the activity of MOF nanozymes at an identical pH of 7.0.

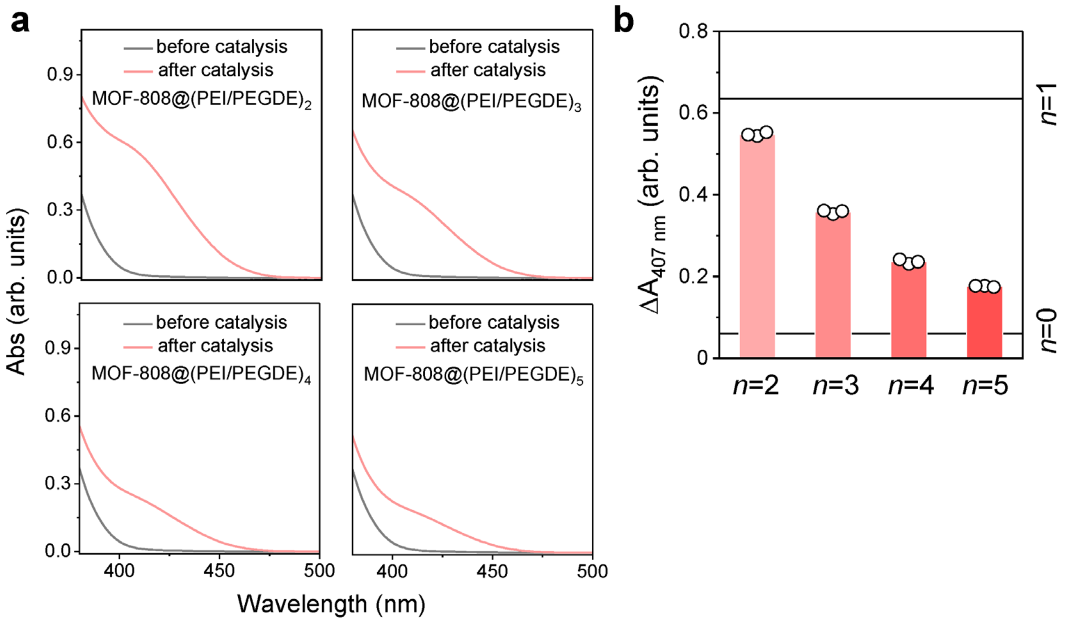

Supplementary Fig. 47. Catalytic hydrolysis of pNPP by MOF-808@(PEI/PEGDE)*ₙ* NPs. (a) UV–vis spectra of pNPP solutions before and after 20-min incubation with MOF-808@(PEI/PEGDE)$_n$ NPs, n = 2, 3, 4, and 5. (b) Absorbance changes at 407 nm ($\Delta A_{407\ nm}$) corresponding to panel a. $\Delta A_{407\ nm}$ for n = 0 and 1 are also shown for comparison. Data in (b) are expressed as mean values ± SEM, n = 3.

We studied the cycles of the PEI confinement process on the activity improvement. Up to five cycles of PEI incubation and PEGDE crosslinking were performed, and the morphology and XRD patterns of the MOF-808 nanozymes remained unchanged (Supplementary Fig. 45). However, when the PEI confinement cycle increases from one to five, the activity decreases (Supplementary Fig. 47). This might be due to the restriction of pNPP diffusion and that too many hydroxyl ions lead to lowered hydrolytic activity.

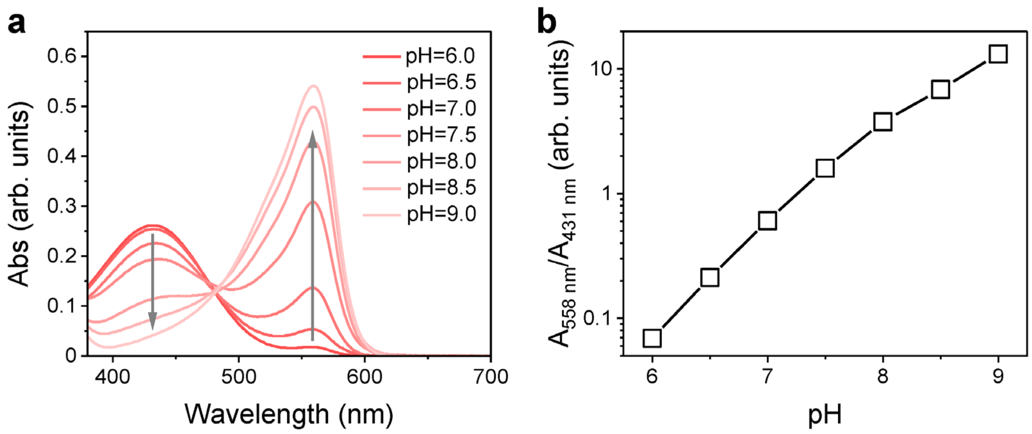

Supplementary Fig. 48. pH indication by phenol red. (a) UV–vis spectra of 10 μg phenol red in 1.6 mL buffer at different pHs. (b) Plots of ratios of the absorbance at 558 nm ($A_{558\ nm}$) to that at 431 nm ($A_{431\ nm}$) versus pH values corresponding to panel a.

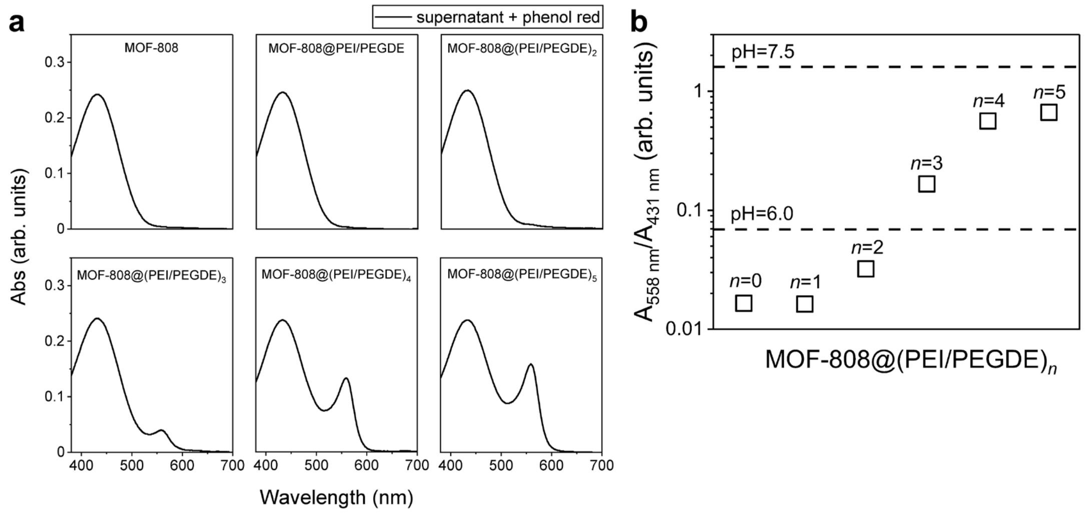

Supplementary Fig. 49. pH indication of the supernatant of MOF-808@(PEI/PEGDE)$_n$ NPs. (a) UV–vis spectra of 1.6 mL of the supernatant of MOF-808@(PEI/PEGDE)$_n$ NPs in the presence of 10 μg phenol red, $n = 0, 1, 2, 3, 4$, and 5. (b) Plots of ratios of absorbance at 558 nm to absorbance at 431 nm ($A_{558\ nm}/A_{431\ nm}$) corresponding to panel a.

As shown in Supplementary Fig. 49, one cycle of the PEI confinement process does not change the pH of the bulk solution. When more than two cycles were employed, we observed the pH changes of the bulk solutions. Considering that MOF-808@PEI/PEGDE NPs exhibit the highest activity (Supplementary Fig. 47), we could conclude that PEI confinement modulates the microenvironmental pH, leading to the enhanced activity.

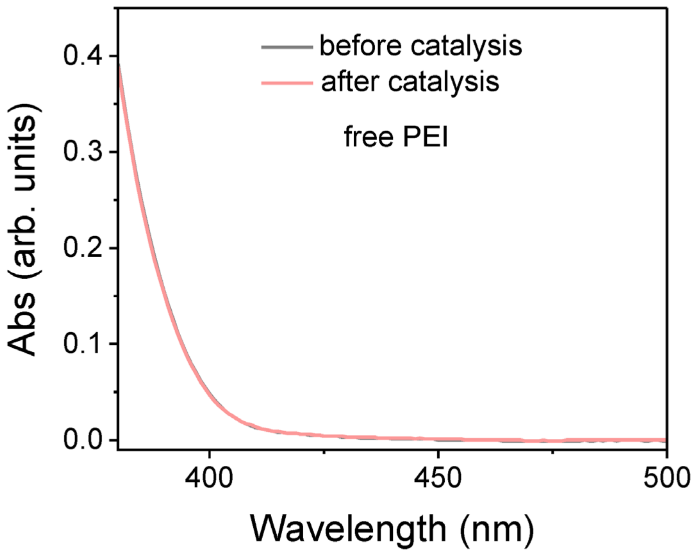

Supplementary Fig. 50. UV–vis spectra of pNPP solutions before and after 20-min incubation with free PEI. PEI concentration was 5.8 μg/mL.

The content of PEI coupled with PEGDE of MOF-808@PEI/PEGDE NPs is around 2.9%, which means that around 5.8 μg comes from the confined polymer in 200 μg NPs. Although we were not able to obtain the accurate content of PEI, we estimated that the PEI amount is less than 2.9%. We directly employed 5.8 μg/mL free PEI to catalyse the hydrolysis of pNPP. As expected, we did not observe any hydrolytic activity of PEI.

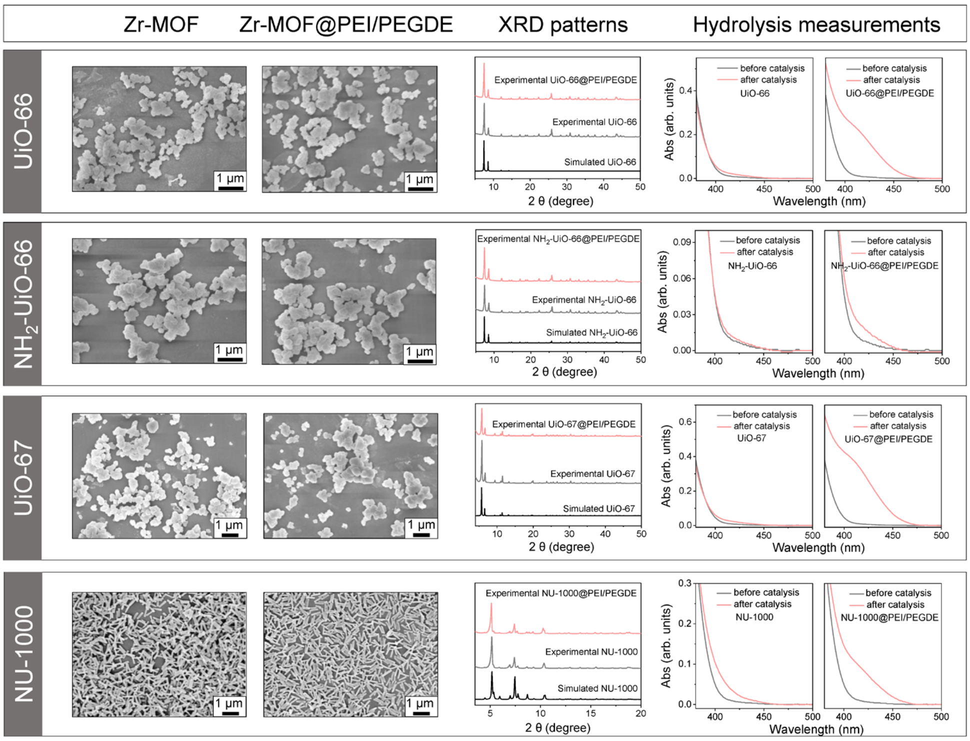

Supplementary Fig. 51. Improved hydrolase-like activity of other Zr-MOF NPs through PEI confinement. Representative SEM images, XRD patterns, and hydrolysis measurements of Zr-MOF NPs and Zr-MOF@PEI/PEGDE NPs, including UiO-66 NPs, NH_2-UiO-66 NPs, UiO-67 NPs, and NU-1000 NPs. SEM images and XRD patterns were obtained from one batch of NPs.

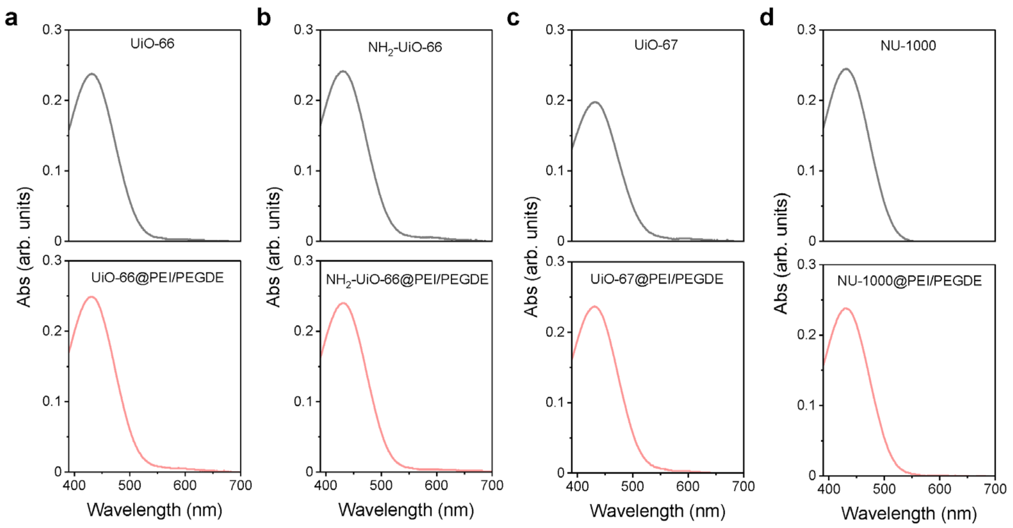

Supplementary Fig. 52. pH indication of the supernatant of Zr-MOF NPs by phenol red. (a) UV–vis spectra of 10 μg phenol red in 1.6 mL of the supernatant of (a) UiO-66 NPs and UiO-66@PEI/PEGDE NPs, (b) NH_2-UiO-66 NPs and NH_2-UiO-66@PEI/PEGDE NPs, (c) UiO-67 NPs and UiO-67@PEI/PEGDE NPs, and (d) NU-1000 NPs and NU-1000@PEI/PEGDE NPs.

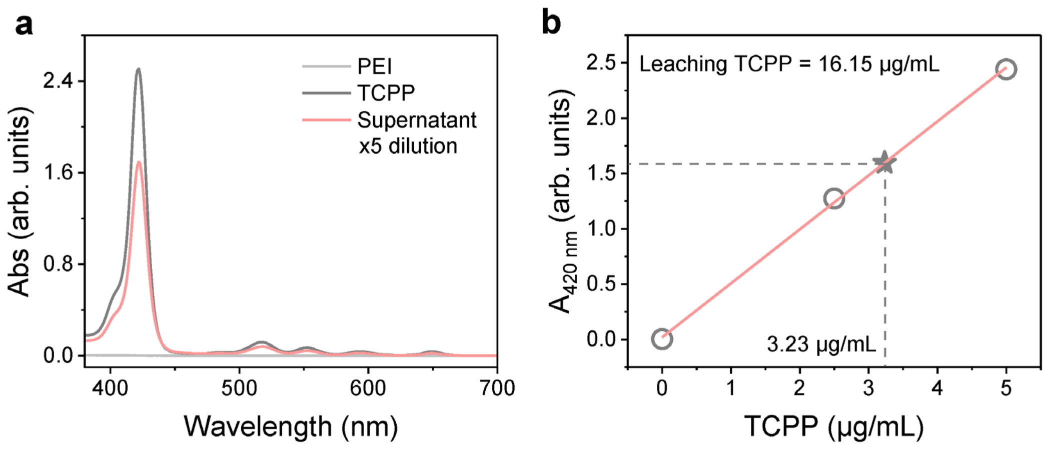

Supplementary Fig. 53. Influence of PEI incubation on TCPP leaching in PCN-222 NPs. (a) UV–vis spectra of 20 mg/mL PEI, 5 μg/mL TCPP, and the diluted supernatant of 10 mg/mL PCN-222 NPs after incubation with 20 mg/mL PEI. (b) Standard curve of TCPP in ethanol containing 20 mg/mL PEI. The calculated leaching TCPP was 16.15 μg/mL according to the standard curve.

We further incubated PCN-222 NPs in PEI to monitor the possible release of TCPP ligand. We observed an absorbance signal of TCPP in the supernatant after PEI incubation, which proved the release of TCPP (Supplementary Fig. 53a). To determine the concentration of the released TCPP, we plotted a standard curve of TCPP in PEI solution. The leached TCPP was calculated to be about 16.15 μg/mL (Supplementary Fig. 53b). It was noted that the employed concentration of PCN-222 NPs was 10 mg/mL, and the overall TCPP concentration in PCN-222 NPs was about 6.58 mg/mL. Hence, the released TCPP accounted for only 0.25% of the overall TCPP in PCN-222 NPs.

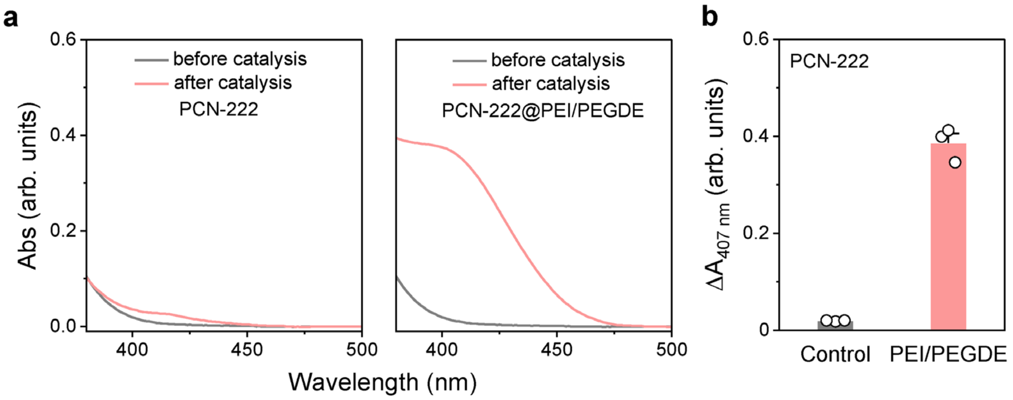

Supplementary Fig. 54. PEI confinement increases the hydrolase-like activity of PCN-222 NPs. (a) UV–vis spectra of bis(4-nitrophenyl) phosphate (bNPP) solutions before and after 20-min incubation with PCN-222 NPs, and PCN-222@PEI/PEGDE NPs. (b) Absorbance changes at 407 nm ($\Delta A_{407\ nm}$) corresponding to panel a. Data in (b) are expressed as mean values ± SEM, n = 3.

We monitored the hydrolase-mimicking activity of PCN-222 NPs before and after PEI modification, observing a significant activity improvement following PEI modification (Supplementary Fig. 54). Therefore, we excluded that the release of 0.25% TCPP resulted in the activity enhancement of PCN-222 NPs after PEI incubation.

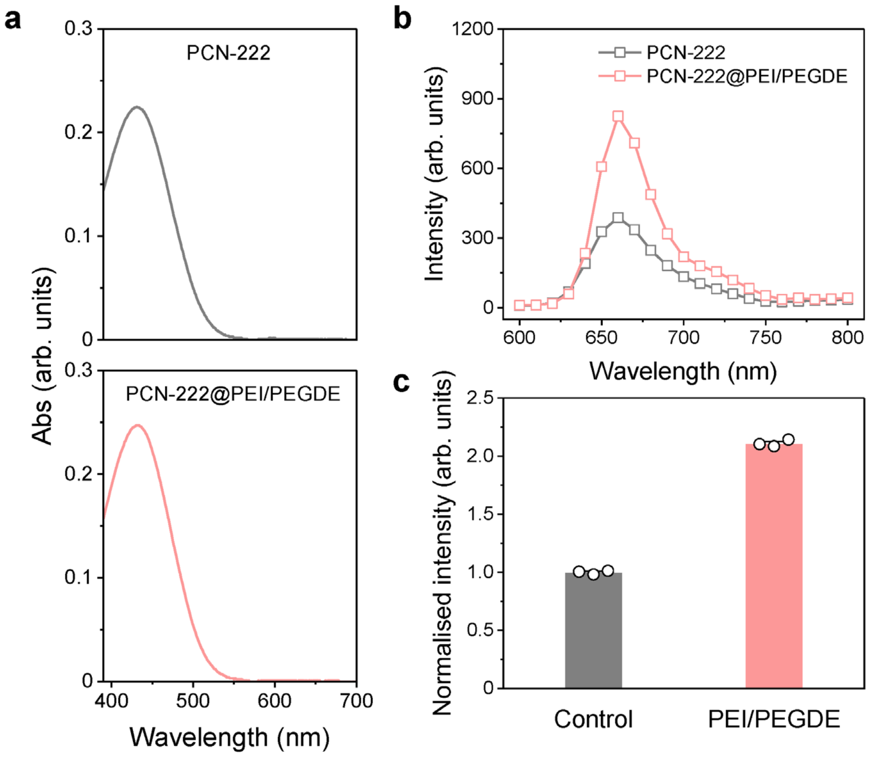

Supplementary Fig. 55. PEI confinement achieves alkaline microenvironment. (a) UV–vis spectra of 10 µg phenol red (a basic pH indicator) in 1.6 mL of the supernatant of PCN-222 NPs and PCN-222@PEI/PEGDE NPs. (b) Fluorescence emission spectra of 20 µg/mL PCN-222 NPs and PCN-222@ PEI/PEGDE NPs in water. $\lambda_{ex} = 420$ nm. (c) Normalised fluorescence intensities at 660 nm corresponding to panel b. Data in (c) are expressed as mean values ± SEM, $n = 3$.

We further found that PEI modification did not change the bulk pH, as indicated by the basic pH indicator phenol red (Supplementary Fig. 55a). Instead, we observed an increase in the fluorescence intensity of PCN-222 NPs after PEI modification, which demonstrated that PEI increased the local pH within PCN-222 NPs (Supplementary Fig. 55b-c). The activity enhancement of PCN-222 NPs through PEI modification could be ascribed to the alkaline microenvironment.

Supplementary Tables

Supplementary Table 1. Summary of BET surface areas of the nanozymes.

Nanozymes	BET surface areas (m^2/g)
PCN-222-Fe	1725
PCN-222-Fe@PAA	1285
MOF-808	1320
MOF-808@PEI/PEGDE	875

Supplementary Table 2. Calculated binding strength between PAA and different ligands.

System	Binding energy (kcal/mol)
PAA/H_3O^+	−48.3
PAA/$Tris^+$	−21.1
PAA/oxTMB	−0.5

Supplementary References

1. Feng, D. et al. Zirconium–metalloporphyrin PCN-222: mesoporous metal–organic frameworks with ultrahigh stability as biomimetic catalysts. *Angew. Chem. Int. Ed.* **51**, 10307–10310 (2012).

2. Zhang, Y., Tsitkov, S. & Hess, H. Proximity does not contribute to activity enhancement in the glucose oxidase-horseradish peroxidase cascade. *Nat. Commun.* **7**, 13982 (2016).

3. Wang, S. et al. DNA-functionalized metal–organic framework nanoparticles for intracellular delivery of proteins. *J. Am. Chem. Soc.* **141**, 2215–2219 (2019).

4. Fan, K. et al. Optimization of Fe_3O_4 nanozyme activity via single amino acid modification mimicking an enzyme active site. *Chem. Commun.* **53**, 424–427 (2016).

5. Gao, X. et al. Controllable synthesis of a smart multifunctional nanoscale metal–organic framework for magnetic resonance/optical imaging and targeted drug delivery. *ACS Appl. Mater. Interfaces* **9**, 3455–3462 (2017).

6. Teranishi, T., Hosoe, M., Tanaka, T. & Miyake, M. Size control of monodispersed Pt nanoparticles and their 2D organization by electrophoretic deposition. *J. Phys. Chem. B* **103**, 3818–3827 (1999).

7. Baek, J. et al. Bioinspired metal–organic framework catalysts for selective methane oxidation to methanol. *J. Am. Chem. Soc.* **140**, 18208–18216 (2018).

8. Katz, M. J. et al. A facile synthesis of UiO-66, UiO-67 and their derivatives. *Chem. Commun.* **49**, 9449–9451 (2013).

9. Zhao, M. et al. Selective epitaxial growth of oriented hierarchical metal–organic framework heterostructures. *J. Am. Chem. Soc.* **142**, 8953–8961 (2020).

10. Zhang, X., Yang, Q., Lang, Y., Jiang, X. & Wu, P. Rationale of 3,3′,5,5′-Tetramethylbenzidine as the Chromogenic Substrate in Colorimetric Analysis. *Anal.Chem.* **92**, 12400–12406 (2020).

11. Zierath, D. L., Hassett, J. J., Banwart, W. L., Wood, S. G. & Means, J. C. Sorption of benzidine by sediments and soils. *Soil Sci.* **129**, 277–281 (1980).

12. Ebara, M., Hoffman, J. M., Hoffman, A. S., Stayton, P. S. & Lai, J. J. A photoinduced nanoparticle separation in microchannels via pH-sensitive surface traps. *Langmuir* **29**, 5388–5393 (2013).

13. Chen, G. & Hoffman, A. S. Graft copolymers that exhibit temperature-induced phase transitions over a wide range of pH. *Nature* **373**, 49–52 (1995).
